# Biocompatible Mesoporous Materials for Bone Therapy

**DOI:** 10.1002/advs.202508740

**Published:** 2025-08-27

**Authors:** Biao Yu, Yan Wu, Yang Hong, Peng Wang, Xiaowen Liu, Yonghui Deng, Jiacan Su

**Affiliations:** ^1^ Institute of Translational Medicine Shanghai University Shanghai 200444 P. R. China; ^2^ MedEng‐X Institutes Shanghai University Shanghai 200444 P. R. China; ^3^ Musculoskeletal Organoid Research Center Shanghai University Shanghai 200444 P. R. China; ^4^ National Center for Translational Medicine (Shanghai) SHU Branch Shanghai University Shanghai 200444 P. R. China; ^5^ School of Medicine Shanghai University Shanghai 200444 P. R. China; ^6^ Department of Orthopedics Xinhua Hospital Shanghai Jiao Tong University School of Medicine Shanghai 200092 P. R. China; ^7^ Department of Chemistry Shanghai Key Laboratory of Molecular Catalysis and Innovative Materials and State Key Laboratory of Coatings for Advanced Equipment iChEM Fudan University Shanghai 200433 P. R. China

**Keywords:** bone regeneration, bone repair, bone therapy, drug delivery system, mesoporous materials

## Abstract

As global population aging intensifies, bone‐related diseases have become a significant public health challenge, necessitating the development of efficient and precise bone repair strategies. Biocompatible mesoporous materials (BMMs), characterized by their high specific surface area, tunable pore size, multidimensional functionalization potential, and excellent biocompatibility, have demonstrated revolutionary application prospects in treating bone‐related diseases. This review systematically summarizes the latest research progress and application mechanisms of BMMs (e.g., mesoporous silica, mesoporous bioactive glass) in bone‐related diseases. It also thoroughly discusses the synthesis methods and functionalization strategies of BMMs, including designs for biocompatibility and biodegradability, targeting capabilities, bio‐responsiveness, and regulation of cellular behavior. The article emphasizes and analyzes the preclinical research applications of BMMs in bone‐targeted drug delivery, bone tissue engineering, bone implant coatings, and integrated diagnosis and therapy. Finally, it provides a retrospective analysis and future perspectives on the application of BMMs in bone‐related diseases, aiming to promote a transition from fragmented breakthroughs to systematic development in BMMs research. This effort seeks to provide a scientific framework for optimizing material design and advancing clinical applications.

## Graphical abstract

Biocompatible mesoporous materials (BMMs), characterized by high specific surface area, tunable pore size, different shapes and structures, and multidimensional functionalized modifications, have revolutionary applications in treating bone‐related diseases. This review systematically summarizes the synthesis methods and functionalization strategies of BMMs, focusing on preclinical research applications for bone‐related diseases, including bone‐targeted drug delivery, bone tissue engineering, bone implant coating, and theranostics, and provides an outlook for future research.

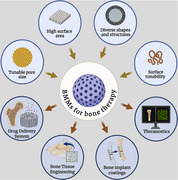

## 1 Introduction

The accelerating process of global population aging has made bone‐related diseases one of the most severe public health challenges of the 21st century.^[^ ^1^, ^2^, ^3^ ^]^ According to data from the International Osteoporosis Foundation, ∼500 million people worldwide suffer from osteoporosis, with postmenopausal women accounting for 30%–50% of cases, and individuals aged 55 and older experience up to 37 million fragility fractures.^[^ ^4^, ^5^, ^6^ ^]^ Of particular concern is the fact that critical‐sized bone defects caused by trauma, tumor resection, or osteomyelitis are difficult to heal naturally, posing a serious threat to patients' quality of life.^[^ ^7^ ^]^ Current clinical treatment strategies face dual dilemmas: for metabolic bone diseases (e.g., osteoporosis), systemic drug administration lacks bone‐targeting specificity, resulting in low drug bioavailability. Additionally, long‐term use of bisphosphonate drugs may induce complications such as osteonecrosis of the jaw.^[^ ^8^, ^9^ ^]^ Although autologous bone grafting is considered the gold standard for structural bone defects, it carries a donor site morbidity rate of up to 20%. Allogeneic bone grafting, meanwhile, has an immunorejection reaction incidence exceeding 30%, while artificial bone implants commonly exhibit delayed osseointegration and long‐term loosening risks.^[^ ^10^, ^11^, ^12^ ^]^ These limitations have driven researchers to explore innovative breakthroughs in biomaterials science.

In this context, the development of bone repair materials has undergone a paradigm shift from biomimetic simulation to functional integration. First‐generation biomaterials, such as 316L stainless steel and titanium alloys, focused on mechanical adaptability but were limited by the stress shielding effect and the issue of permanent foreign body residue.^[^ ^13^, ^14^ ^]^ Second‐generation bioactive materials, including hydroxyapatite and β‐tricalcium phosphate, exhibited osteoconductive properties; however, their low porosity (typically <50%) hindered cell infiltration.^[^ ^15^, ^16^ ^]^ In contrast, third‐generation intelligent biomaterials aim to achieve structural‐functional integration, with biocompatible mesoporous materials (BMMs) emerging as a standout candidate due to their unique mesoscale characteristics, such as pore sizes ranging from 2 to 50 nm and specific surface areas exceeding 500 m^2^g^−1^.^[^ ^17^ ^]^ BMMs are regarded as one of the most promising platform materials for clinical translation in bone repair.^[^ ^18^, ^19^, ^20^, ^21^ ^]^ The breakthrough advantages of BMMs lie in their multidimensional regulation capabilities. At the mesoscale, the abundant pore network not only provides sufficient space for drug loading but also mimics the mechanical properties of bone tissue, offering adequate mechanical performance.^[^ ^22^ ^]^ Furthermore, its topological structure can potentially guide mesenchymal stem cell migration through fluid shear stress regulation.^[^ ^23^, ^24^ ^]^ At the molecular scale, the surface‐rich silanol groups or modified functional groups (e.g., amino groups) enable chemical coupling of drugs or growth factors. Combined with external stimuli or internal pH/enzyme‐responsive controlled release mechanisms, this allows for intelligent control of local drug concentrations.^[^ ^25^, ^26^ ^]^ Most importantly, the dynamic degradation properties of BMMs in bone tissue engineering scaffolds can be coordinated with the deposition rate of newly formed bone tissue. This addresses the risk of collapse caused by premature degradation of traditional bio‐ceramics, presenting a promising solution to this critical challenge.

**Figure 1:**
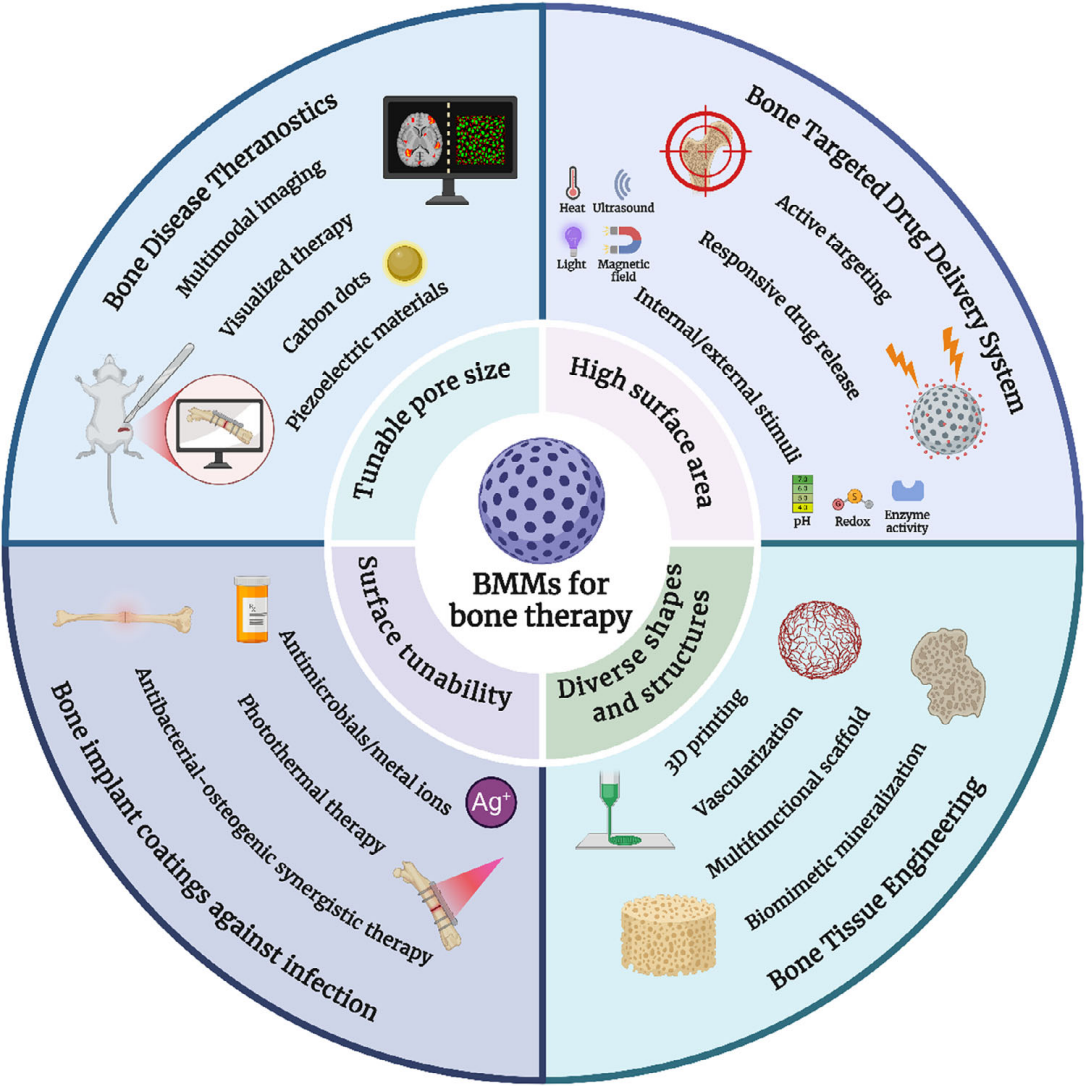
BMMs for bone therapy. BMMs, featuring large specific surface areas, tunable pore sizes, shapes, structures, and surface functionalization capabilities, are widely employed in bone‐targeted drug delivery systems, bone tissue engineering, anti‐infection coatings for bone implants, and bone disease theranostics. Created with BioRender.com.

Specifically, as shown in **Figure** **1**, BMMs show great potential in four key areas for bone‐related diseases: bone‐targeted drug delivery, bone tissue engineering, bone implant coatings, and theranostics. Intelligent responsive systems constructed using BMMs (e.g., pH‐, light‐, or magnetic‐responsive systems) enable precise drug release regulation in bone‐targeted drug delivery.^[^ ^20^, ^27^ ^]^ These systems facilitate the synergistic delivery of anti‐resorption and pro‐anabolic agents (e.g., bisphosphonates and parathyroid hormone), significantly enhancing the efficacy of osteoporosis treatment. In bone tissue engineering, BMMs mimic the natural bone microenvironment through biomimetic mineralization mechanisms (e.g., directional growth of hydroxyapatite) and multifunctional scaffold designs (e.g., mesoporous bioactive glass (MBG) composites with hydrogels).^[^ ^28^, ^29^, ^30^ ^]^ By integrating 3D printing technology, BMMs enable the customization of scaffolds with mechanical adaptability and vascularization capabilities, thereby promoting cell adhesion, differentiation, and bone regeneration.^[^ ^31^ ^]^ Addressing the challenge of bone implant‐associated infections, BMMs coatings loaded with antimicrobial agents (e.g., antibiotics or metal ions) or integrated with photothermal therapy demonstrate highly efficient antibacterial properties. Additionally, these coatings achieve antibacterial‐osteogenic synergy through releasing metal ions and delivering growth factors or drugs.^[^ ^32^ ^]^ Furthermore, BMMs‐based integrated diagnosis and therapy systems for bone diseases incorporate multimodal imaging (e.g., magnetic resonance imaging (MRI) and fluorescence (FL)), as well as visualized therapy.^[^ ^33^, ^34^ ^]^ For instance, mesoporous polydopamine nanoprobes hold promise for MRI‐visualized intra‐articular drug delivery, offering a novel paradigm for precision medicine.^[^ ^35^ ^]^

Despite the immense potential of BMMs in diagnosing and treating bone‐related diseases, their research remains in its early stages, lacking systematic summaries and evaluations. Current literature predominantly focuses on studies involving individual materials or specific application domains, with a paucity of comprehensive reviews on the application of mesoporous materials in bone repair, particularly regarding their functionalization strategies and systematic summaries of preclinical research findings. Therefore, how to optimize the design of mesoporous materials and further enhance their efficacy in bone repair has become a critical question in contemporary research. This review aims to systematically summarize the research progress of BMMs in bone repair, focusing on exploring their functionalization methods and underlying mechanisms of action in bone repair. The article will revisit the current achievements of mesoporous materials in bone repair applications, analyze the challenges, and propose future research directions. Through these summaries and perspectives, we hope to provide theoretical support and practical guidance for the further application of mesoporous materials in bone repair, while offering insights for future research endeavors.

## 2 Fundamentals of BMMs

### 2.1 Overview of BMMs

Mesoporous materials refer to materials with abundant pore structures and pore sizes ranging from 2 to 50 nanometers.^[^ ^36^, ^37^ ^]^ They possess the following characteristics: rich porous structure, high specific surface area, tunable pore size, large pore volume, diverse morphology, and adjustable surface property.^[^ ^38^, ^39^ ^]^ First, the porous structure endows them with efficient molecular sieving capabilities and excellent stability, enabling mesoporous materials to perform exceptionally well in molecular sieving, catalysis, and drug delivery.^[^ ^40^, ^41^, ^42^ ^]^ Second, the high specific surface area can typically reach 500–1500 m^2^g^−1^ and even higher. The advantage of a high specific surface area lies in its ability to provide a larger adsorption capacity and improve drug loading.^[^ ^21^ ^]^ A larger specific surface area can offer more active sites in catalysis and adsorption processes, thereby accelerating reaction rates. Third, their pore size can be controlled by adjusting synthesis conditions, such as templates, reaction temperature, and reaction time.^[^ ^26^ ^]^ Mesoporous materials with different pore sizes can be selected according to specific needs for loading and delivering molecules of various sizes. For example, smaller pore sizes are suitable for drug molecules, while larger pore sizes are appropriate for loading larger biomolecules such as proteins and genes. The tunability of pore size and wall properties also allows for precise control of drug release rates in drug delivery, achieving on‐demand release effects.^[^ ^43^ ^]^ Mesoporous materials can selectively adsorb different molecules based on pore size and wall properties, which is commonly used in gas adsorption and molecular sieving applications.^[^ ^44^ ^]^ Fourth, their large pore volume helps accommodate many guest molecules. In applications such as drug delivery and catalytic reactions, higher pore volume allows more drugs or catalysts to load, enabling longer drug release durations or catalytic activity.^[^ ^45^, ^46^, ^47^ ^]^ Fifth, using different surfactants can obtain mesoporous materials with various structural materials, such as rod‐like, plate‐like, hollow, and core‐shell structures.^[^ ^48^ ^]^ Finally, the surface property of mesoporous materials can be chemically modified to introduce various functional groups, enhancing their interactions with molecules, cells, or other biological entities.^[^ ^49^ ^]^

**Figure 2:**
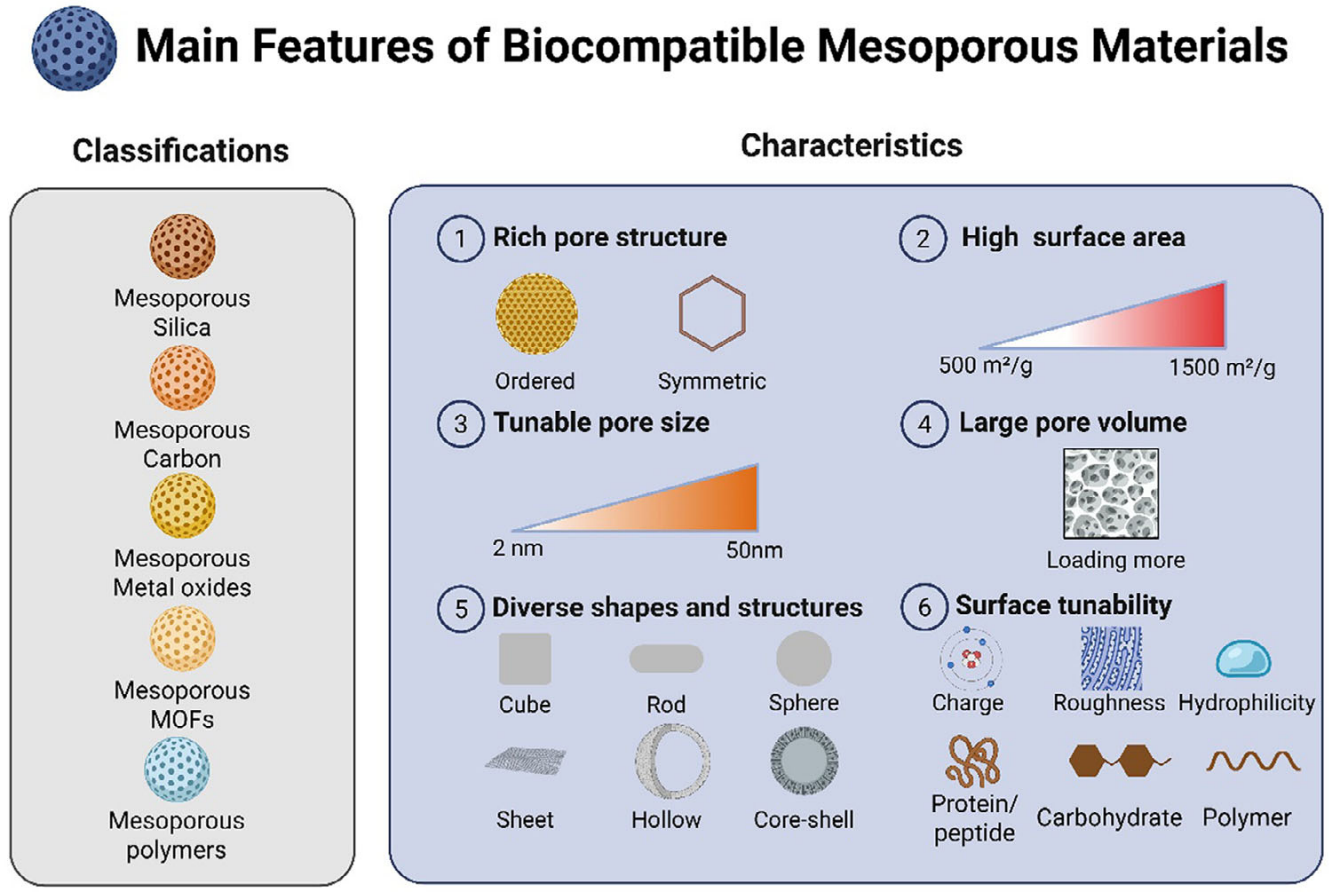
Categories and main features of BMMs. BMMs primarily include mesoporous silica, carbon, metal oxides, metal‐organic frameworks, and mesoporous polymers. BMMs typically feature well‐defined porosity characteristics (e.g., ordered and symmetrical structures), tunable pore sizes, high specific surface areas, large pore volumes, diverse morphologies and architectures (including tubular, rod‐like, spherical, plate‐like, hollow, and core‐shell configurations), along with adjustable surface physicochemical properties (such as charge, roughness, and hydrophilicity) that can be functionally modified with biomolecules (proteins/peptides, carbohydrates, polymers). Created with BioRender.com.

BMMs refer to materials that possess the characteristics of mesoporous materials and exhibit excellent biocompatibility within biological systems (**Figure** **2**). The biocompatibility of these materials enables them to interact well with surrounding biological tissues and cells, allowing them to remain in biological environments for extended periods without causing significant immune responses, toxicity, or rejection reactions.^[^ ^50^ ^]^ Furthermore, these materials typically do not cause harm to the organism. BMMs primarily include mesoporous silica materials (e.g., MCM‐41, SBA‐15),^[^ ^51^, ^52^, ^53^ ^]^ mesoporous carbon materials (e.g., MCM‐48, CMK‐3),^[^ ^54^, ^55^, ^56^ ^]^ mesoporous metal oxides (e.g., ZnO, ZrO_2_),^[^ ^57^, ^58^, ^59^ ^]^ mesoporous metal‐organic frameworks (MOFs),^[^ ^60^, ^61^ ^]^ mesoporous hydroxyapatite,^[^ ^62^, ^63^, ^64^ ^]^ mesoporous calcium phosphate,^[^ ^65^, ^66^ ^]^ mesoporous bioactive glass,^[^ ^67^, ^68^, ^69^ ^]^ biodegradable polymer‐based mesoporous materials (such as mesoporous polydopamine),^[^ ^70^, ^71^ ^]^ and some mesoporous materials formed by the self‐assembly of natural or synthetic organic molecules.^[^ ^72^ ^]^ The applications of BMMs are mainly focused on drug delivery, tissue engineering, wound repair, gene carriers, and bioimaging.

### 2.2 Preparation Methods of BMMs

The commonly employed synthesis methods for BMMs encompass soft‐template and hard‐template approaches, each exhibiting distinct advantages and limitations.^[^ ^73^ ^]^ Through optimization of reaction conditions and process parameters, precise control over pore structure, specific surface area, pore volume, and degradation properties of mesoporous materials can be effectively achieved. As the most prevalent technique for mesoporous material preparation, the soft‐template method finds extensive application in synthesizing mesoporous silica, carbon, and metal oxides.^[^ ^74^, ^75^, ^76^ ^]^ Its fundamental principle relies on utilizing organic molecules such as ionic surfactants, block copolymers, or star‐shaped copolymers as templating agents to construct mesoporous channels.^[^ ^77^, ^78^ ^]^ These templating agents, consisting of hydrophilic and hydrophobic domains, can self‐assemble into solution micelles. Framework precursors interact with these micelles through hydrogen bonding, electrostatic forces, or conjugation effects, co‐assembling to form mesoporous architectures.^[^ ^79^ ^]^ This method demonstrates advantages, including straightforward synthesis procedures and diverse templating agent selection. The assembly behavior can be modulated by adjusting the reaction system's solution polarity, pH, and ionic strength, enabling effective regulation of mesoporous channel structure and dimensions.^[^ ^80^ ^]^ Furthermore, the inherent instability of organic molecules allows facile removal of residual templating agents through extraction or calcination.

Depending on specific reaction conditions, soft‐template methods can be categorized into sol‐gel processes, hydrothermal synthesis, and evaporation‐induced self‐assembly (EISA). The sol‐gel method involves dissolving templating agents in solvents, followed by pH adjustment to facilitate precursor hydrolysis. After system stabilization, framework precursors undergo hydrolysis and crosslinking polymerization.^[^ ^81^, ^82^ ^]^ The framework material co‐assembles with templating agents to form ordered mesostructures during this process. As crosslinking progresses, the product gradually precipitates as a gel, with subsequent template removal yielding mesoporous materials.^[^ ^83^ ^]^ Hydrothermal synthesis shares similarities with sol‐gel processes but typically occurs in autoclaves at elevated temperatures (80–300 °C) with extended reaction durations.^[^ ^84^, ^85^ ^]^ The EISA technique proves particularly suitable for synthesizing lamellar or membrane‐like mesoporous materials.^[^ ^86^, ^87^ ^]^ Its mechanism relies on gradual solvent evaporation that increases concentrations of templating agents and framework precursors beyond the critical micelle concentration, inducing liquid crystal phase formation. Framework cross‐linking then fixes this phase to produce ordered mesoporous products. Solvent selection constitutes a crucial factor in EISA, requiring balanced component solubility and appropriate volatility to prevent prolonged synthesis times.

The hard‐template method, alternatively termed nanocasting, employs rigid mesoporous materials (e.g., silica or carbon) with predefined pore structures as synthetic guides, beneficial for producing mesoporous metal oxides and carbons.^[^ ^88^, ^89^ ^]^ These chemically inert templates function solely as physical barriers during synthesis. Guest materials infiltrate template pores via capillary forces, followed by precursor solidification through calcination and subsequent template removal to obtain final products.^[^ ^90^ ^]^ This versatile approach enables the synthesis of ordered mesostructures where direct control of precursor hydrolysis/cross‐linking proves challenging. However, limitations include restricted template variety, pore dimension/shape regulation difficulties, and complex synthesis procedures. Beyond templated approaches, alternative strategies include self‐templating and template‐free methods. Self‐templating exploits defects or voids generated during material growth/etching processes,^[^ ^91^, ^92^ ^]^ while template‐free methods utilize rigid ligand‐supported frameworks.^[^ ^93^ ^]^

Building on this foundation of precisely engineered mesostructures, the inherent physicochemical properties of BMMs—tailored through controlled synthesis protocols—establish the essential canvas for advanced functionalization. As detailed in Section 2.3, these architecturally defined systems become strategic platforms for implanting biological intelligence. This progression from structural control to biological function embodies the core design philosophy driving next‐generation bone regenerative scaffolds.

### 2.3 Functionalization of BMMs

Despite the potential and intriguing properties of BMMs in the biomedical field, particularly in bone tissue repair, a single physical property is insufficient to meet the demands of specific applications. Therefore, functionalization design of mesoporous materials is essential to enhance their performance further and broaden their applications. The core of functionalization design lies in modifying the surface or internal structure of mesoporous materials to endow them with specific physical, chemical, or biological functions, thereby improving their performance in diagnostic and therapeutic applications. This includes designs for biocompatibility and biodegradability, targeted delivery, bio‐responsive properties, and regulation of cellular behavior.

#### 2.3.1 Design for Biocompatibility and Biodegradability

Biocompatibility and biodegradability are key characteristics for applying mesoporous materials in the biomedical field, especially in drug delivery, tissue engineering, and bone repair. Through functionalization design, the compatibility of mesoporous materials with the biological system can be effectively enhanced, and their safe degradation in the body can be ensured.^[^ ^50^ ^]^ Biocompatibility design aims to strengthen the affinity of mesoporous materials with the biological system, reduce immune responses and cytotoxicity, and ensure their long‐term safe use in the body. Standard methods include: 1) Surface amination: The introduction of amino groups (–NH_2_) can enhance the hydrophilicity and cellular compatibility of the materials.^[^ ^94^, ^95^, ^96^ ^]^ Lam et al. demonstrated that amino‐functionalized mesoporous MOFs can enhance biocompatibility.^[^ ^60^ ^]^ BMSCs co‐cultured with HMUiO‐66‐NH2 at concentrations up to 80 µg mL^−1^ for 7 days did not show significant cytotoxicity. 2) Surface coating: Coating the material with a biocompatible polymer (such as polyethylene glycol) can reduce non‐specific interactions with the immune system, decreasing the host's rejection response. For instance, Goel et al. reported that polyethylene glycol‐modified mesoporous silica nanoparticles (PEGylated MSNs) are more stable and exhibit better immune evasion in vivo, leading to longer circulation times.^[^ ^97^ ^]^ The blood circulation half‐life (t1/2) of PEGylated MSN is estimated to be ≈4 h, which is significantly longer than that of the same sample without a PEG protective coating (t1/2 < 10 min). 3) Natural biomolecule modification: For example, surface modification with natural biomolecules such as fibronectin can improve biocompatibility and promote cell growth and adhesion.^[^ ^98^ ^]^ Cheng et al. also reported that macrophage membrane‐coated MSNs loaded with selenium did not show significant cytotoxicity at concentrations as high as 10 µgmL^−1^.^[^ ^99^ ^]^

The design of biodegradability aims to enhance the ability of the material to gradually degrade over time within the body, releasing harmless products and thereby avoiding long‐term accumulation or adverse reactions. Studies have shown that altering parameters such as pore size and porosity, which affect the contact area between the framework of mesoporous materials and water molecules, can be used to control the degradability of mesoporous materials.^[^ ^50^ ^]^ For example, bare MSNs are more degradable than non‐porous silica nanoparticles, and spherical bare MSNs degrade faster than rod‐shaped MSNs.^[^ ^100^, ^101^ ^]^ In addition to surface area, the surface functionalization of mesoporous materials has been proven to affect biodegradability by promoting hydration or hydrolysis processes. Coatings significantly impact the degradability of mesoporous materials, but specific coatings within each category can produce different outcomes. Kim et al. demonstrated that coating MSNs with polyethyleneimine (PEI‐MSN) accelerates hydrolytic degradation.^[^ ^102^ ^]^ The total degradation of PEI‐MSN increased rapidly over time, reaching a maximum degradation of 81% on day 7, which was significantly higher than the total degradation of bare MSN (68%). Cauda et al. found that hydrophilic polymer coatings such as PEG significantly slow down the degradation of MSNs in simulated body fluid, depending on the polymer's coverage density and molecular weight.^[^ ^103^ ^]^ Longer and denser polymer shells are more efficient in slowing down the bio‐degradation kinetics than the unfunctionalized mesoporous nanoparticles. Moreover, different functional group modifications also affect the biodegradability of mesoporous materials. Phenyl‐functionalized MSNs degrade significantly faster, followed by chloropropyl and aminopropyl‐functionalized MSNs.^[^ ^104^ ^]^ In phosphate‐buffered saline, amine‐functionalized MSNs exhibit the fastest degradation behavior, followed by carboxylated MSNs and non‐functionalized MSNs.^[^ ^105^ ^]^ Finally, altering the chemical composition of the mesoporous material framework can regulate and control its degradation rate, such as incorporating metal oxides. Studies have reported that doping with manganese, calcium, iron, copper, magnesium, and zinc can modulate the degradation behavior of MSNs.^[^ ^106^, ^107^, ^108^, ^109^, ^110^ ^]^ Mesoporous organosilica nanoparticles with disulfide bonds in their hybrid structure are conducive to enhanced biodegradability.^[^ ^111^, ^112^ ^]^

#### 2.3.2 Design for Targeting

Targeting design enables mesoporous materials to recognize and target specific cells or tissues, thereby enhancing therapeutic efficacy and reducing damage to healthy cells. This strategy is critical in drug delivery, gene therapy, and cancer treatment. By modifying the surface of mesoporous materials with specific biomolecules, the materials can specifically bind to target cells or tissues. Standard targeting designs include: 1) Peptide modifications: Acidic peptides can confer the ability to target bone tissue to the carrier, such as aspartic acid oligopeptide (Asp_n_) and glutamic acid oligopeptide (Glu_n_), which are rich in carboxyl (─COOH) groups.^[^ ^113^, ^114^ ^]^ The enrichment rates of DAsp8‐modified MSN in rat femurs and vertebrae 24 h after intravenous injection were three times and two times higher than those of unmodified MSN, respectively. These carboxyl groups can chelate with calcium ions (Ca^2^⁺) on the hydroxyapatite surface in bone tissue. By specifically binding to hydroxyapatite, acidic peptides can deliver drugs or therapeutic molecules to bone tissue. This targeting ability not only increases the concentration of the drug in the bone tissue but also reduces the distribution of the drug in non‐target tissues, thereby reducing systemic side effects. 2) Antibody or antigen modification: Conjugating monoclonal antibodies or antigens to the surface of mesoporous materials endows them with the ability to specifically recognize tumor cells or pathogens, which is used for targeted therapy and immunotherapy. For example, Goel et al. conjugated anti‐CD105 antibodies to PEGylated MSNs, endowing the nanoconjugates with tumor‐targeting capabilities.^[^ ^97^ ^]^ In the targeted group, nanoparticle uptake increased to 11.4 ± 2.1% ID g^−1^ six hours after administration, whereas in the non‐targeted group, it reached 3.6 ± 0.3% ID g^−1^. 3) Surface glycosylation: Modifying the surface with sugar molecules allows the materials to specifically bind to receptors on the cell surface (such as glycoproteins and glycolipids). This design can enhance the affinity of the materials for immune cells or specific cell types. For example, Brevet et al. reported that mannosylated MSNs significantly improved photodynamic therapy efficiency against breast cancer cells.^[^ ^115^ ^]^ Ma et al. utilized hyaluronic acid‐conjugated MSNs (MSNs‐HA) to enable more efficient uptake by CD44‐positive cancer cells through receptor‐mediated endocytosis.^[^ ^116^ ^]^ The green fluorescence of MSNs‐HA was uniformly distributed throughout the cytoplasmic region of HeLa cells after 3 h of incubation. However, in L929 and MCF‐7 cells (which express lower levels of HA receptors), green fluorescence was barely detectable even after 12 h of incubation with MSNs at concentrations as high as 200 mg mL^−1^.

#### 2.3.3 Design for Responsiveness

Responsive design refers to the ability of mesoporous materials to respond to changes in the microenvironment within the biological system, such as pH, temperature, and enzyme activity, thereby regulating their structure, function, or drug release properties. Many pathological microenvironments, such as those in tumors and osteoporosis, exhibit lower pH values.^[^ ^114^ ^]^ Drug release or structural changes can be triggered in specific pH environments by designing pH‐responsive mesoporous materials.^[^ ^117^ ^]^ Common pH‐responsive design strategies include: 1) Modification with weakly acidic or basic groups: For example, introducing amino or carboxyl groups can cause structural changes in the material under acidic or basic conditions, thereby regulating drug release or material solubility. Xiao et al. constructed a pH‐responsive carrier by grafting polycations onto anionic carboxylate‐modified SBA‐15 through ionic interactions.^[^ ^118^ ^]^ The polycations are closed gates, storing doxorubicin (DOX) within the mesopores. When the ionized carboxyl groups are protonated in response to pH changes, the polycations detach from the surface, releasing DOX from the mesopores. Within 12 h, only ≈20% of DOX was released in acidic solution (pH = 2.0), whereas in a weakly alkaline environment (pH = 7.6), rapid and substantial DOX release (≈64%) was observed within 12 h. 2) pH‐sensitive polymer composites: For instance, pH‐sensitive materials such as polyvinyl alcohol and polystyrene‐sulfonate are combined with mesoporous materials, enabling the material to swell or contract under different pH conditions. Temperature‐responsive materials have critical applications in biomedicine, such as temperature‐controlled drug release and cancer treatment. Combining temperature‐sensitive polymers (such as poly(N‐isopropylacrylamide, PNIPAM) with mesoporous materials allows the material to transition at a specific temperature, regulating drug release.^[^ ^119^ ^]^ At the same temperature for 10 days, the drug release amount of L3‐PNIPAm gel is more than four times the silicified L3 gels. Enzyme‐responsive design leverages the activity of specific endogenous enzymes to trigger material degradation or drug release, holding critical importance in targeted tumor therapy due to the overexpression of certain enzymes – such as matrix metalloproteinases and phospholipases – within the tumor microenvironment.^[^ ^120^, ^121^ ^]^ A typical implementation involves capping mesoporous materials with enzyme‐sensitive components like phospholipids, which act as gating mechanisms that open enzymatically to release the payload. Recently, Fang et al. developed an enzyme‐responsive formulation using doxorubicin‐loaded, small‐sized mesoporous silica nanoparticles (DMSNs) encapsulated within nanoliposomes.^[^ ^122^ ^]^ The liposomal membrane incorporated 1,2‐dipalmitoyl‐sn‐glycero‐3‐phospho‐rac‐(1‐glycerol) (DPPG) and 1,2‐dipalmitoyl‐sn‐glycero‐3‐phosphocholine (DPPC), phospholipids sensitive to secretory phospholipase A2 in human colorectal tumors. Incubation in phospholipase‐mimicking solution for 45 min resulted in 90% drug release, whereas the enzyme‐free control group exhibited minimal release.

#### 2.3.4 Design for Cell Behavior Regulation

The functionalization of mesoporous materials enables the delivery of drugs or molecules and, more importantly, allows for regulating cell behavior, presenting unique advantages in tissue engineering and regenerative medicine. This design primarily focuses on the synergistic effects of multiple dimensions, including cell adhesion, proliferation, and differentiation. Regarding cell adhesion and proliferation, mesoporous materials significantly enhance cell adhesion capacity, proliferation rate, and migration characteristics through surface modification or binding with extracellular matrix molecules (such as collagen and fibronectin).^[^ ^60^ ^]^ Specifically, surface peptide modification (e.g., the Arg‐Gly‐Asp (RGD) peptides) can effectively enhance the binding force between cells and the material, promoting the adhesion and growth of specific cells such as osteoblasts and chondrocytes.^[^ ^123^ ^]^ Moreover, by mimicking the components of the extracellular matrix (e.g., collagen and silk fibroin), mesoporous materials further optimize the cell proliferation environment, providing an ideal scaffold for cell culture in tissue engineering. It is worth noting that mesoporous materials can also scavenge reactive oxygen species (ROS) by doping with metal oxides (e.g., CeO_2_, MnO_2_, CuO, TaO) to modulate the pathological microenvironment.^[^ ^124^, ^125^ ^]^ In terms of cell differentiation and induction, mesoporous materials successfully guide the differentiation process of cells through surface modification with growth factors, drugs, or signaling molecules. For example, in bone repair, mesoporous materials loaded with bone morphogenetic proteins and other growth factors effectively promote the directed differentiation of stem cells into osteoblasts.^[^ ^22^ ^]^

## 3 Applications of BMMs in Bone Therapy

### 3.1 Bone Targeted Drug Delivery System

**Figure 3:**
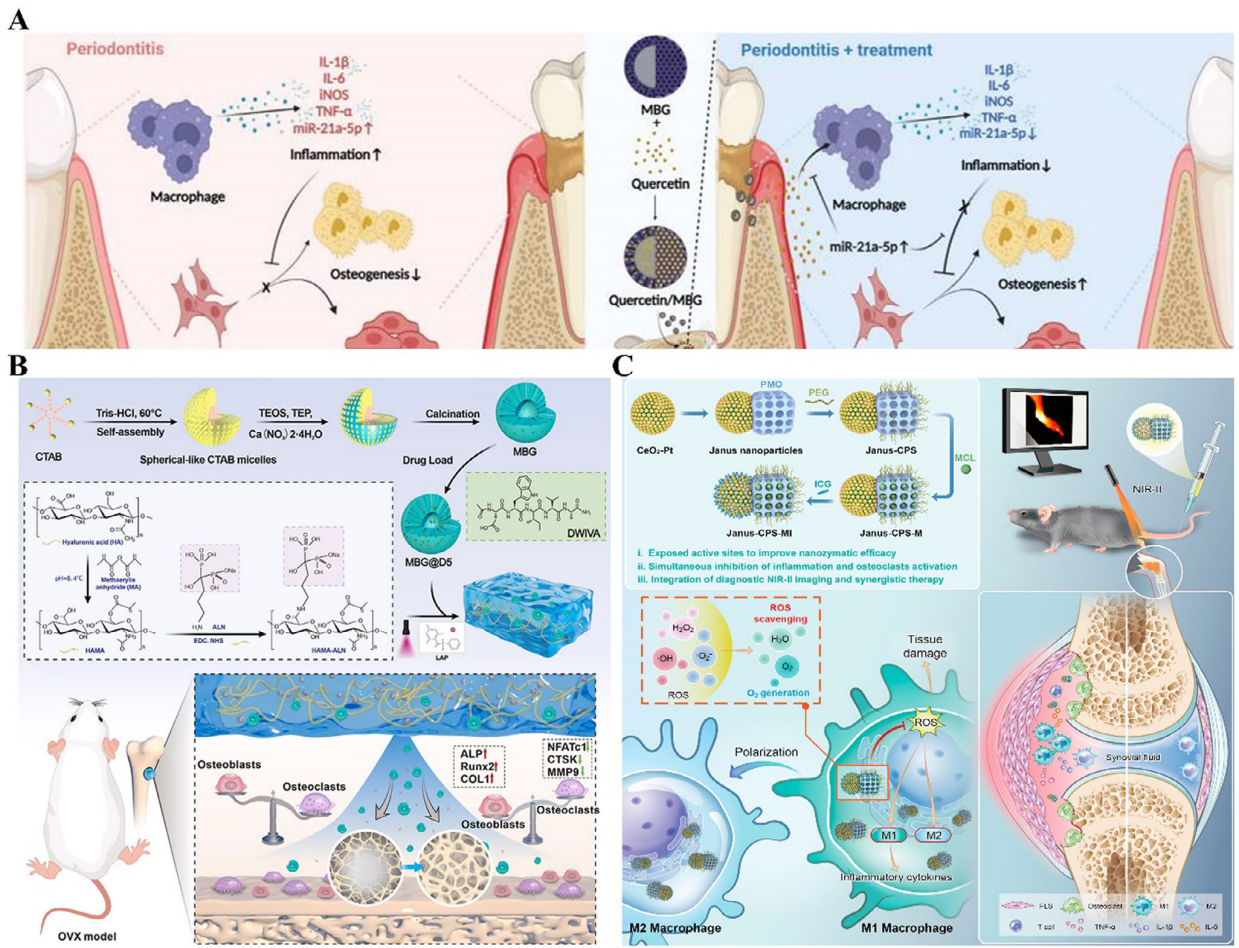
A) Schematic illustration of the mesoporous nanodelivery system containing quercetin to reconstruct alveolar bone defects in periodontitis. Reproduced under the terms of the Creative Commons CC BY license.^[^ ^126^ ^]^ Copyright 2024, The Authors. B) Composite hydrogel drug delivery system doped with mesoporous bioactive glass synergistically modulates osteoblast and osteoclast activity to promote repair of osteoporotic bone defects. Reproduced under the terms of the Creative Commons CC BY‐NC‐ND license.^[^ ^127^ ^]^ Copyright 2025, The Authors. C) Schematic illustration of the synthesis of the Janus nanoplatform and its capability of early diagnosis and synergistic treatment of rheumatoid arthritis. Reproduced with permission.^[^ ^131^ ^]^ Copyright 2023, American Chemical Society.

In recent years, drug delivery systems (DDS) have made significant progress in the medical field, especially in achieving precise spatiotemporal controlled release of drugs. Among them, BMMs are often used to develop DDS for bone‐related diseases due to their open mesoporous channels, high specific surface area, ease of surface modification, and multifunctional integration. These materials include mesoporous bioactive glass, silica, hydroxyapatite, and calcium phosphate nanomaterials.^[^ ^20^ ^]^ Drugs are commonly loaded within the pores and can be adsorbed on the mesoporous materials' surface through electrostatic interactions. The drugs that can be delivered include natural small‐molecule drugs, bioactive molecules, and nucleic acid molecules. For example, a drug delivery system based on quercetin‐loaded MBG has the function of sustained quercetin release. Compared with pure MBG treatment, it can better promote bone regeneration in bone defects and modulate the immune microenvironment (**Figure** **3**A).^[^ ^126^ ^]^ The composite hydrogel drug delivery system with MBG loaded with the bone‐enhancing peptide DWIVA (D5, Asp‐Trp‐Ile‐Val‐Ala) and the osteoclastogenesis‐inhibiting drug alendronate (ALN) synergistically modulates osteoblast and osteoclast activities to promote the repair of osteoporotic bone defects (Figure 3B).^[^ ^127^ ^]^ Bone‐targeted MSNs have been used to deliver antibiotics to treat osteomyelitis caused by Staphylococcus aureus.^[^ ^128^ ^]^ Thavasyappan Thambi et al. also reported chitosan‐coated MSN loaded with salmon calcitonin for oral treatment of osteoporosis.^[^ ^129^ ^]^ In addition, Janus‐structured mesoporous materials have become an up‐and‐coming platform due to their unique dual‐sided properties.^[^ ^130^ ^]^ These properties enable the materials to integrate multiple functions to maximize therapeutic effects. For example, Dong et al. developed a Janus nanoplatform, one side of which is composed of CeO_2_−Pt nanozyme subunits, and the other side consists of periodic mesoporous organosilica (PMO) loaded with magnolol, for synergistic anti‐osteoclast and anti‐inflammatory treatment of rheumatoid arthritis (Figure 3C).^[^ ^131^ ^]^

**Figure 4:**
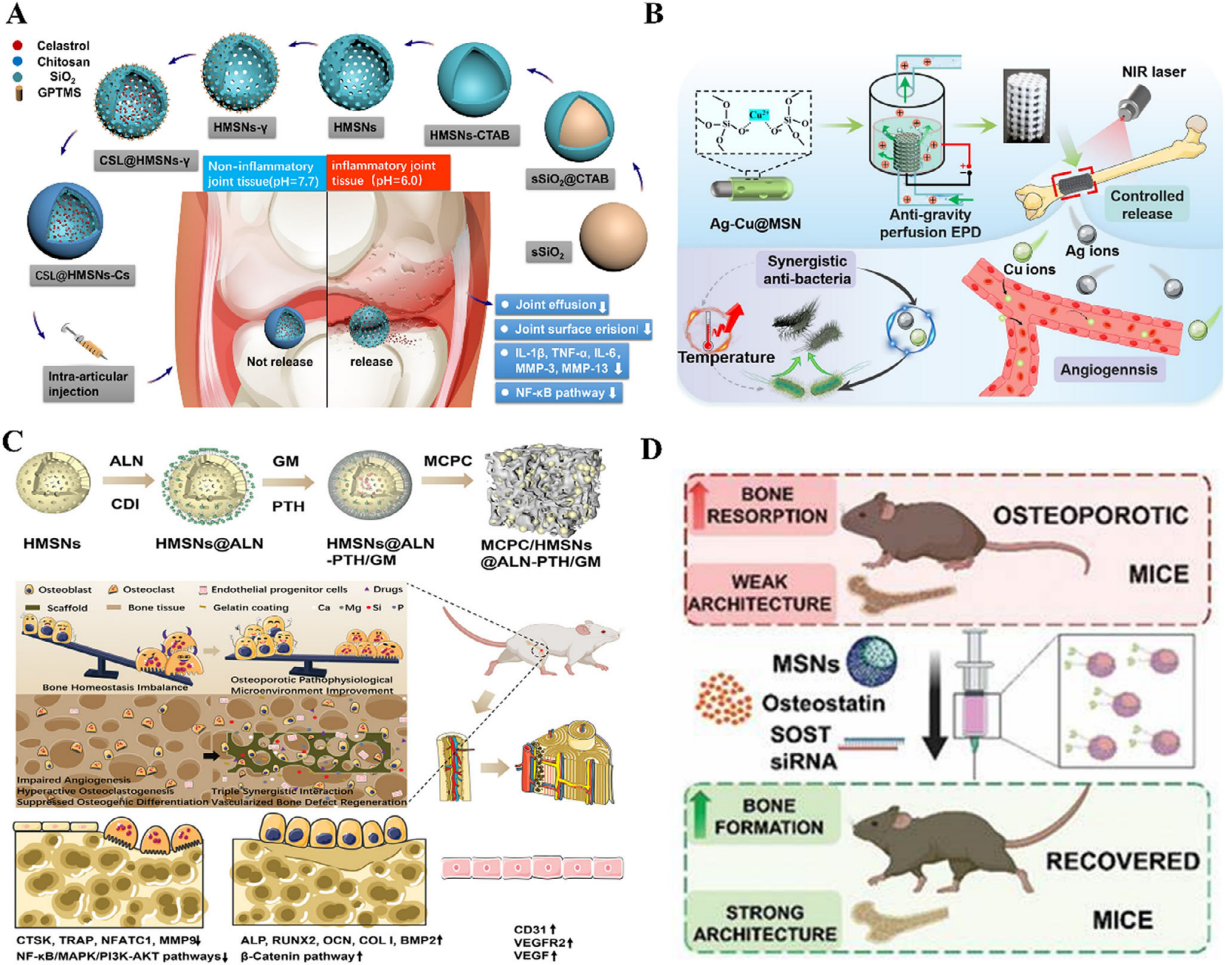
A) Schematic of MSNs loaded with celastrol as a pH‐responsive drug delivery system for anti‐inflammatory treatment of osteoarthritis. Reproduced under the terms of the Creative Commons CC BY license.^[^ ^133^ ^]^ Copyright 2020, The Authors. B) Schematic illustration of NIR photothermally triggered antimicrobial coating with metal ion release. Reproduced with permission.^[^ ^135^ ^]^ Copyright 2024, Elsevier. C) Schematic of the synthesis of MCPC/HMSNs@ALN‐PTH/GM composites and their effect on the triple synergistic effects of osteoporotic bone defect regeneration. Reproduced with permission.^[^ ^139^ ^]^ Copyright 2023, John Wiley and Sons. D) Schematic diagram of MSNs delivering SOST siRNA and osteoclastin in combination for the treatment of osteoporosis. Reproduced under the terms of the Creative Commons CC BY license.^[^ ^140^ ^]^ Copyright 2021, The Authors.

Intelligent responsive DDS based on BMMs represents a significant breakthrough in drug delivery, capable of triggering drug release under specific physiological or pathological conditions. These systems are particularly suitable for treating bone‐related diseases with unique microenvironments, such as osteoporosis and osteoarthritis. pH‐responsive DDS utilizes the pH gradient changes inherent in biological processes to trigger drug release under acidic conditions.^[^ ^27^ ^]^ For instance, MSNs loaded with isoliquiritigenin (ISL) have been designed for acid‐responsive drug release in the microenvironment of osteoporosis, effectively inhibiting osteoclast activity and reducing bone resorption.^[^ ^132^ ^]^ MSNs loaded with celastrol have been developed to respond to pH changes in the osteoarthritis microenvironment, thereby releasing drugs to alleviate inflammation (**Figure** **4**A).^[^ ^133^ ^]^ Enzyme‐responsive designs utilize the activity of specific enzymes in vivo to induce degradation of the material or drug release. Poly‐L‐glutamic acid (PG)‐coated MSN‐Ag exhibits responsive Ag ion release via bacterial secretion of glutamyl ribonucleic acid endonuclease for bone tissue infections.^[^ ^134^ ^]^ In addition to internal stimuli, external physical stimuli, such as light, ultrasound, magnetic fields, and heat, are also commonly used in developing BMM‐based intelligent responsive DDS for treating bone‐related diseases. For example, chitosan‐coated silver and copper co‐doped MSN nanoparticles exhibit near‐infrared light (NIR) photothermally‐triggered release of metal ions, enhancing bone implants' antimicrobial and osteogenic effects (Figure 4B).^[^ ^135^ ^]^ MBG nanocarriers doped with chlorin e6 have been employed for NIR‐triggered smart release of chlorin e6 in bone tumor therapy.^[^ ^136^ ^]^ A light‐responsive anti‐inflammatory drug delivery platform for diclofenac has been constructed using azobenzene‐modified MSNs and β‐cyclodextrin‐modified poly(2‐methacryloyloxyethyl phosphorylcholine), exploiting their supramolecular interactions, for the treatment of osteoarthritis.^[^ ^137^ ^]^ Furthermore, multi‐stimuli‐responsive DDS based on BMMs have been developed to enhance the controlled release of drugs. For instance, Zhao et al. developed a dual‐stimulus (pH and potential) responsive drug release system based on MSNs as a coating for titanium implants.^[^ ^138^ ^]^ The release of ibuprofen from the hydrogel on the titanium plate responds to pH changes and occurs significantly slower than the release from naked MSNs, showing nearly linear release kinetics. Applying an external potential can modulate the ibuprofen release profile; for example, using a high negative voltage accelerates the release of ibuprofen.

Multifunctional DDS based on BMMs achieves synergistic effects by co‐delivering two or more therapeutic agents, significantly enhancing the efficacy of bone‐related disease treatments. In osteoporosis therapy, these systems typically involve the combined delivery of anti‐resorptive agents (e.g., bisphosphonates) and anabolic agents (e.g., parathyroid hormone (PTH), bone morphogenetic protein‐2 (BMP‐2)).^[^ ^141^ ^]^ Combining anti‐resorptive and anabolic agents enhances bone repair and regeneration, reducing systemic side effects. For instance, Zhao et al. utilized MSNs to co‐deliver alendronate (a bisphosphonate) and PTH. This system inhibited osteoclast activity via alendronate while stimulating osteoblast differentiation through PTH (Figure 4C).^[^ ^139^ ^]^ The results demonstrated that synergistic co‐delivery markedly improved bone repair and reduced systemic toxicity compared to monotherapy. Similarly, He et al. employed BMP‐2 peptides and dexamethasone (DEX)‐loaded MSNs to synergistically enhance the osteogenic differentiation of bone marrow mesenchymal stem cells (BMSCs).^[^ ^142^ ^]^ In bone tumor treatment, such systems often require simultaneously eliminating residual cancer cells and promoting bone regeneration. For example, Liu et al. developed manganese (Mn)‐doped MBG loaded with chlorin e6 for combined bone tumor therapy and regeneration.^[^ ^136^ ^]^ BMM‐based multifunctional delivery systems also enable synergistic co‐delivery of genes and drugs. Patricia et al., for instance, utilized MSNs to co‐deliver small interfering RNA (siRNA) and osteogenic peptides (e.g., osteostatin), achieving a combinatorial osteoporosis therapy.^[^ ^140^ ^]^ The nanoparticles were first loaded with osteostatin, coated with cationic polymers to bind SOST siRNA, and finally functionalized with alendronate (ALN)‐modified polyethylene glycol (PEG) to enhance stability and enable bone‐targeted co‐delivery of SOST siRNA and osteostatin (Figure 4D). Subcutaneous injection in ovariectomized (OVX) mice significantly promoted bone formation, restoring values nearly equivalent to those of healthy mice. This nanosystem demonstrated superior osteogenic efficacy compared to the current gold standard therapy, free PTH administration.

Developing targeted DDS has opened new avenues for treating bone‐related diseases. These systems provide unprecedented precision and therapeutic outcomes from controlled‐release mechanisms to smart‐responsive platforms. Multifunctional delivery systems highlight the potential of DDS to address future clinical needs. With ongoing research, more innovative solutions are anticipated to emerge, further improving treatment efficacy for osteoporosis and other bone‐related disorders (**Table** **1**).

**Table 1:** BMMs‐based drug delivery systems for bone therapy.

BMMs	Pore size [nm]	Surface area [m^2^ g^−1^]	Drugs and load capacity [µg mg^−1^]	Release rate/days	Refs.
MSNs	3.4	895	SOST siRNA (3 wt%) Osteostatin (10^−4^ M, impregnation)	1.155 µg mg^−1^/2 (Osteostatin)	[140]
MSNs	2.7	480.7	DMOG (3 mg mL^−1^, impregnation)	95%/14	[143]
MSNs	2.7	621.6	BMP‐2 pDNA (0.90) DEX	61%/4 (pDNA) 75.0%/20 (DEX)	[144]
MSNs	15	189.8	HAS2 (420)	69%/0.5	[145]
MSNs	15	/	miR‐10a (0.0162 nmol/mg), IL‐2 (0.45), TGF‐β ( 0.87)	90%/50 (miR‐10a) 90%/12 (IL‐2 /TGF‐β)	[146]
MSNs	/	299.1	DS (17.4)	59.8%/3	[147]
MSNs	3‐6	/	sCT (2 mgmL^−1^, impregnation)	31.4%±3.6%/0.35	[129]
MSNs	2.1	191	DOX (40)	35%/0.5 (pH5.0) 55%/0.5 (pH2.0)	[148]
MSNs	4.57	960.3	ISL (10 mg mL^−1^, impregnation)	23.97%±1.35%/2.5 (pH7.4) 42.02%±1.63%/2.5 (pH5.5)	[132]
MSNs	/	/	Ag (0.73%)	22ug/mL/3 (with V8 enzyme) 13 ug/mL/3 (without V8 enzyme)	[134]
HMSNs	2.2	1100	PTH (28.2%) ALN (28.2%)	99%/28 (PTH) 90%/50 (ALN)	[139]
HMSNs	2.4	1006.8	Celastrol (28.2%)	21.7%/0.1 (pH7.7) 68.9%/0.1 (pH6.0)	[133]
MBG	/	/	Quercetin(320.25)	65%/21	[126]
MBG	/	/	Peptide DWIVA (1 mg mL^−1^, impregnation)	20%/9	[127]
MBG	4	>350	Ce6 (39.8%)	29.86% (37 °C) 34.9% (45 °C)	[136]
mPDA	/	/	MnCO (0.05 mg mL^−1^, impregnation)	30%/0.17	[149]
PMO	2.2	465	Micheliolide (33.8)	50%/ 7	[131]
Mesoporous hydroxyapatite	30–50	186.06	Mg^2+^ (30%) Icariin (7.69%)	300 ug/mL/28 (Mg^2+)^ 70%/2.5 (pH7.4, Icariin) 90%/2.5 (pH6.4, Icariin)	[150]

### 3.2 Bone Tissue Engineering

Bone tissue engineering has emerged as a pivotal research direction in the biomedical field in recent years. It aims to develop novel strategies for effective bone repair and regeneration by integrating materials science and biology. Key advancements—including biomimetic mineralization mechanisms, multifunctional scaffold designs, vascularization strategies, and 3D printing technologies—have laid a robust foundation for bone regeneration engineering.

The biomimetic mineralization mechanism is one of the core concepts in bone tissue engineering, inspired by the natural bone formation process. Hydroxyapatite, the primary inorganic component of bone tissue, plays a critical role in bone regeneration. Researchers have recently focused on developing material systems that mimic natural bone mineralization, with mesoporous structured materials gaining significant attention due to their exceptional physicochemical properties. By providing a high specific surface area and tunable pore structures, mesoporous materials serve as ideal templates for the oriented growth of hydroxyapatite. Studies indicate mesoporous materials and their surface chemical properties can profoundly influence hydroxyapatite's crystallization orientation and morphology.^[^ ^151^, ^152^ ^]^ For example, introducing specific functional groups (e.g., phosphate groups) on the surface of mesoporous silica can guide hydroxyapatite to grow along particular directions, forming highly ordered nanostructures.^[^ ^153^ ^]^ This oriented mineralization process not only enhances the biocompatibility of materials but also significantly improves their mechanical properties. Furthermore, interfacial molecular mechanisms are pivotal in biomimetic mineralization. Incorporating osteogenic components (e.g., bisphosphonates) onto mesoporous material surfaces can further regulate hydroxyapatite mineralization and promote bone cell adhesion and differentiation.^[^ ^154^ ^]^ Such multilevel regulatory strategies offer novel insights for developing high‐performance bone regenerative materials.

**Figure 5:**
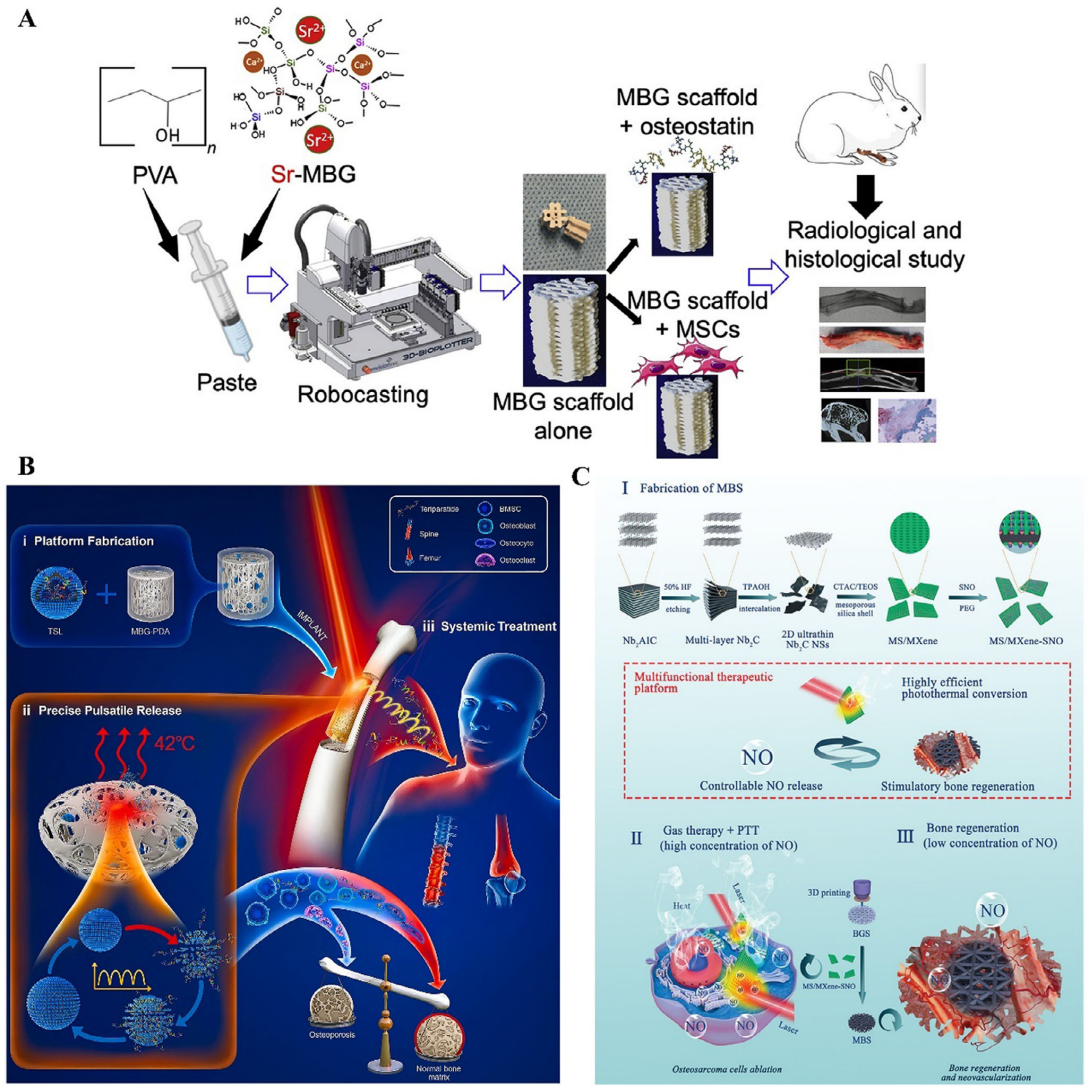
A) Sr‐MBG scaffold loaded with osteoinhibitor for long bone defect repair in rabbit radius. Reproduced under the terms of the Creative Commons CC BY license.^[^ ^29^ ^]^ Copyright 2024, The Authors. B) Schematic diagram of bioactive scaffold with smart pulsatile teriparatide delivery for osteoporotic fracture treatment. Reproduced under the terms of the Creative Commons CC BY‐NC‐ND license.^[^ ^155^ ^]^ Copyright 2022, The Authors. C) Multifunctional scaffolds with features of controllable NO release, highly efficient photothermal conversion, and stimulation of bone regeneration were designed for the diversified treatment of bone tumors. Reproduced with permission.^[^ ^156^ ^]^ Copyright 2020, John Wiley and Sons.

Multifunctional scaffold design represents another critical research direction in bone tissue engineering. Mesoporous bioactive glass (MBG), a material with exceptional bioactivity and degradability, has emerged as an ideal candidate for bone regeneration scaffolds.^[^ ^19^ ^]^ However, regulating cellular behavior through the rational design of scaffold topology remains challenging. Studies have shown that the pore size of MBG scaffolds significantly influences their ability to induce stem cell differentiation.^[^ ^157^ ^]^ By constructing MBG scaffolds with optimized pore structures, researchers can mimic the native bone microenvironment and guide stem cells toward osteogenic differentiation. For instance, MBG scaffolds with 20 nm pores enhance stem cell adhesion, prolong BMP‐2 release, and significantly improve bone regeneration outcomes.^[^ ^158^ ^]^ María Vallet‐Regí et al. also showed that only a certain amount of calcium in MBG upregulates osteogenic differentiation, and that increasing calcium weakens the silica network and thus the osteogenic properties.^[^ ^69^ ^]^ Furthermore, multifunctional scaffold design integrates diverse therapeutic capabilities. In bone defect repair, standalone MBG scaffolds exhibit limited efficacy. Studies demonstrate that MBG scaffolds loaded with osteostatin (an osteoinductive peptide) or bone marrow aspirate enhance bone formation, with osteostatin‐loaded scaffolds achieving superior results (**Figure** **5**A).^[^ ^29^ ^]^ For osteoporotic fracture treatment, incorporating drug molecules (e.g., teriparatide) into MBG scaffolds enables on‐demand release, markedly improving bone remodeling (Figure 5B).^[^ ^155^ ^]^ In bone tumor therapy, MBG scaffolds functionalized with NIR‐II‐triggered photothermal therapy achieve controlled nitric oxide (NO) release: an initial high‐concentration phase exerts antitumor effects. At the same time, subsequent low‐concentration NO synergizes with MBG to enhance angiogenesis and bone regeneration (Figure 5C).^[^ ^156^ ^]^ Additionally, Chen et al. ingeniously hybridised mesoporous piezoelectric SrTiO_3_ nanoparticles loaded with the drug DNA methyltransferase (DNMT) inhibitor epigallocatechin gallate (EGCG) with BG scaffolds to form a multifunctional composite scaffold, achieving efficient PANoptosis immunotherapy for osteosarcoma.^[^ ^159^ ^]^ The increased specific surface area of mesoporous piezoelectric materials not only enhances the efficiency of piezoelectric material sites responding to ultrasound irradiation, thereby promoting sonochemical reactions and ROS generation, but also improves the sensitivity of stress‐responsive drug release. These integrated multifunctional design paradigms offer breakthrough solutions for bone tissue engineering.

The regulation of the osteoimmune microenvironment and bone regeneration represents a current research hotspot in bone tissue engineering. Physical properties of nanomaterials, such as surface morphology, can effectively modulate osteoimmune responses. Xiao et al. reported mesoporous silica nanoparticles with pollen‐like surface morphology (PMSNs) that more effectively suppress macrophage inflammation.^[^ ^163^ ^]^ PMSNs uniquely inhibit macrophage polarization toward pro‐inflammatory phenotypes and improve the osteoimmune microenvironment to promote bone regeneration through physical material‐cell membrane contact. This occurs via upregulation of the cell surface receptor CD28 and inhibition of ERK phosphorylation. Similarly, Ye et al. designed novel mesoporous silica rods (MSR‐CP) whose tapered pore‐conferred surface properties downregulate pro‐inflammatory gene expression and cytokine production in macrophages.^[^ ^164^ ^]^ The immunomodulatory capability of mesoporous bioactive glass (MBG) is likely attributable to its release of multiple bioactive ions. Lai et al. demonstrated that MBG/BSA bone grafts enhance antioxidant production (e.g., SOD, MDA) in inflammatory environments via the IL‐4/STAT6 pathway, promoting macrophage reprogramming toward the M2 phenotype to support osteogenic differentiation and bone regeneration further.^[^ ^165^ ^]^ Liu et al. showed that porous PLGA scaffolds containing 10% MBG elicit faster initial immune responses, facilitating timely macrophage phenotype switching from pro‐inflammatory to anti‐inflammatory.^[^ ^166^ ^]^ This subsequently enhances HUVEC angiogenic potential and BMSC osteogenic capacity by downregulating TNF‐α signaling and upregulating HIF‐1α and PI3K/AKT pathways. Additionally, using BMMs as carriers for immunomodulatory drugs, factors, or metal ions constitutes an effective osteoimmune repair strategy. For instance, Xu et al. reported MBG‐loaded quercetin reshaping the osteoimmune microenvironment and regenerating alveolar bone in periodontitis via the miR‐21a‐5p/PDCD4/NF‐κB pathway.^[^ ^126^ ^]^ At the same time, Guo et al. demonstrated that selenium ion‐loaded MBG clears ROS, regulates macrophage metabolism/polarization, and promotes in vivo bone regeneration.^[^ ^167^ ^]^ These studies reveal novel mechanisms by which BMM surface morphology regulates macrophage immune responses and provide crucial theoretical and experimental foundations for developing new immunomodulatory mesoporous nanomaterials for bone regeneration.

**Figure 6:**
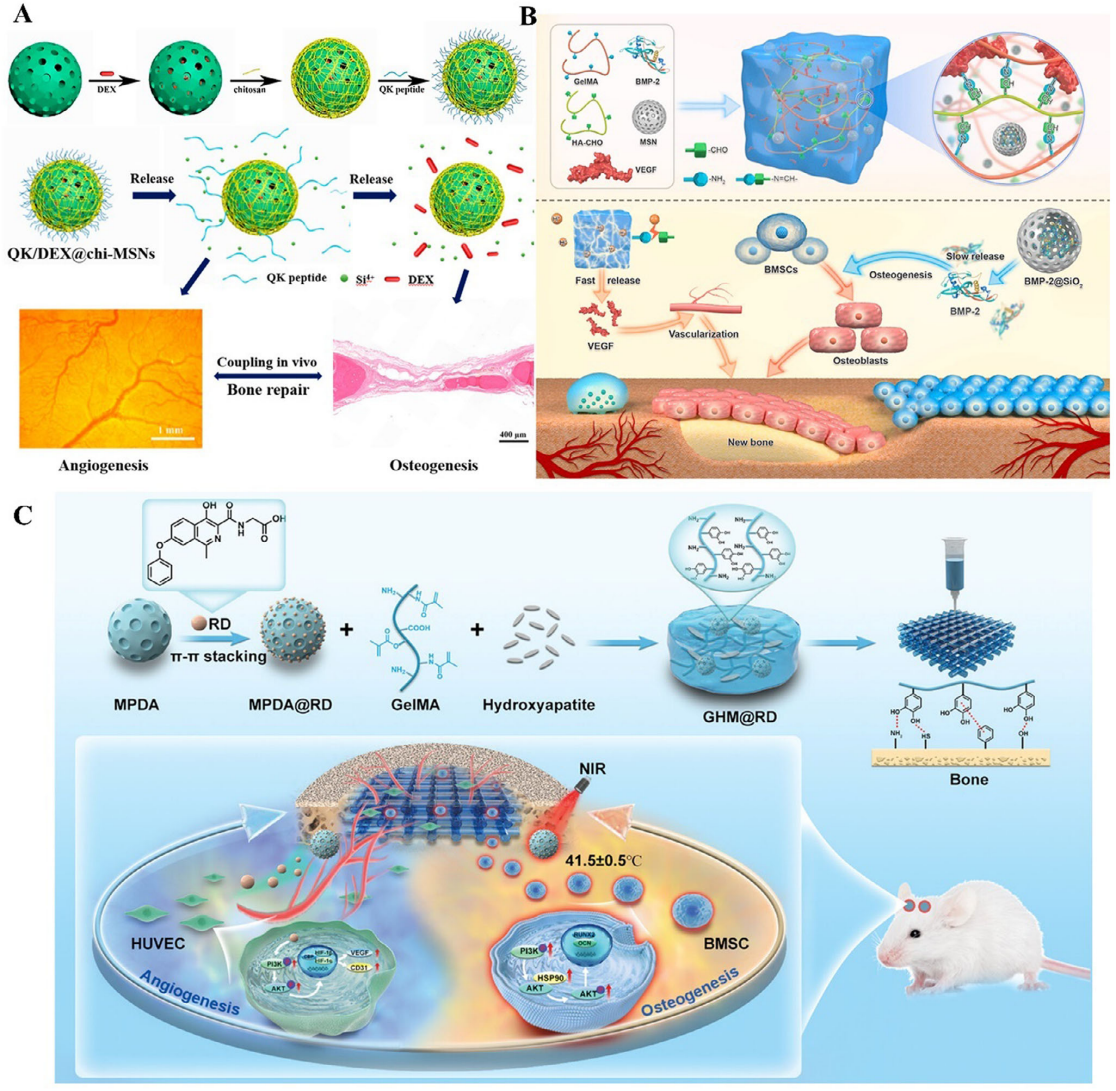
A) Mesoporous silica nanocarriers loaded with angiogenic QK peptide and DEX for accelerated angiogenesis in bone regeneration. Reproduced with permission.^[^ ^160^ ^]^ Copyright 2019, American Chemical Society. B) Schematic illustration of BVHG composite hydrogel for promoting angiogenesis and enhancing osteogenesis. Reproduced under the terms of the Creative Commons CC BY‐NC‐ND license.^[^ ^161^ ^]^ Copyright 2024, The Authors. C) Schematic illustration of the mechanism of vascularized bone regeneration induced by GelMA/HA/MPDA@Roxadustat scaffolds. Reproduced with permission.^[^ ^162^ ^]^ Copyright 2024, John Wiley and Sons.

Vascularization is an indispensable component of bone regeneration. A well‐developed vascular network not only supplies oxygen and nutrients to newly formed bone tissue but also efficiently removes metabolic waste. Researchers have recently focused on developing material systems capable of promoting vascular growth. BMMs, owing to their exceptional drug/ion‐loading capacity and controlled‐release properties, have demonstrated significant potential in vascularization strategies. For instance, Daniel Arcos et al. reported that strontium‐containing MBG promotes bone repair through sustained regeneration of blood vessels.^[^ ^168^ ^]^ Similarly, Saeid Kargozar et al. reported that doping MBG with strontium (Sr) and cobalt (Co) enables dual functionality: Sr promotes bone remodeling, while Co stimulates angiogenesis.^[^ ^169^ ^]^ Vascularized bone regeneration can be accelerated by loading mesoporous materials with vascular endothelial growth factor (VEGF), its mimetic peptides (e.g., QK peptide), and osteogenic drugs. Sun et al., for example, utilized mesoporous silica as a delivery carrier for DEX and the QK peptide (a VEGF mimetic) to achieve rapid vascularized bone regeneration in bone defects (**Figure** **6**A).^[^ ^160^ ^]^ To address the high demand for VEGF during early‐stage vascularization, Cao et al. developed a composite hydrogel for cascade‐controlled growth factor delivery, creating a vascularized and osteogenic microenvironment to enhance bone regeneration (Figure 6B).^[^ ^161^ ^]^ VEGF formed Schiff base bonds with the hydrogel, enabling rapid release in the acidic microenvironment of early‐stage bone injuries to promote angiogenesis. Meanwhile, bone morphogenetic protein‐2 (BMP‐2) encapsulated in MSNs achieved sustained release, facilitating bone repair. The hydrogel released over 80% of VEGF and BMP‐2 in acidic media, significantly higher than in neutral media (≈60%). This synergistic strategy of early VEGF burst release and prolonged BMP‐2 delivery effectively promoted vascularized bone regeneration in rat cranial defects. To further enhance vascular network reconstruction in significant bone defects, You et al. loaded roxadustat (RD)—a compound stabilizing hypoxia‐inducible factor‐1α (HIF‐1α) under normoxic conditions—onto mesoporous polydopamine nanoparticles (MPDA) (Figure 6C).^[^ ^162^ ^]^ These nanoparticles were mixed with GelMA/hyaluronic acid (HA) hydrogel bioink to fabricate multifunctional 3D‐printed scaffolds (GHM@RD). In vitro results demonstrated that under mild photothermal stimulation, GHM@RD scaffolds activated the PI3K/AKT/HSP90 pathway in BMSCs and the PI3K/AKT/HIF‐1α pathway in human umbilical vein endothelial cells (HUVECs), achieving coupled angiogenic‐osteogenic effects. In vivo experiments confirmed that RD and mild photothermia synergistically induced early vascularization and bone regeneration in critical‐sized defects.

**Figure 7:**
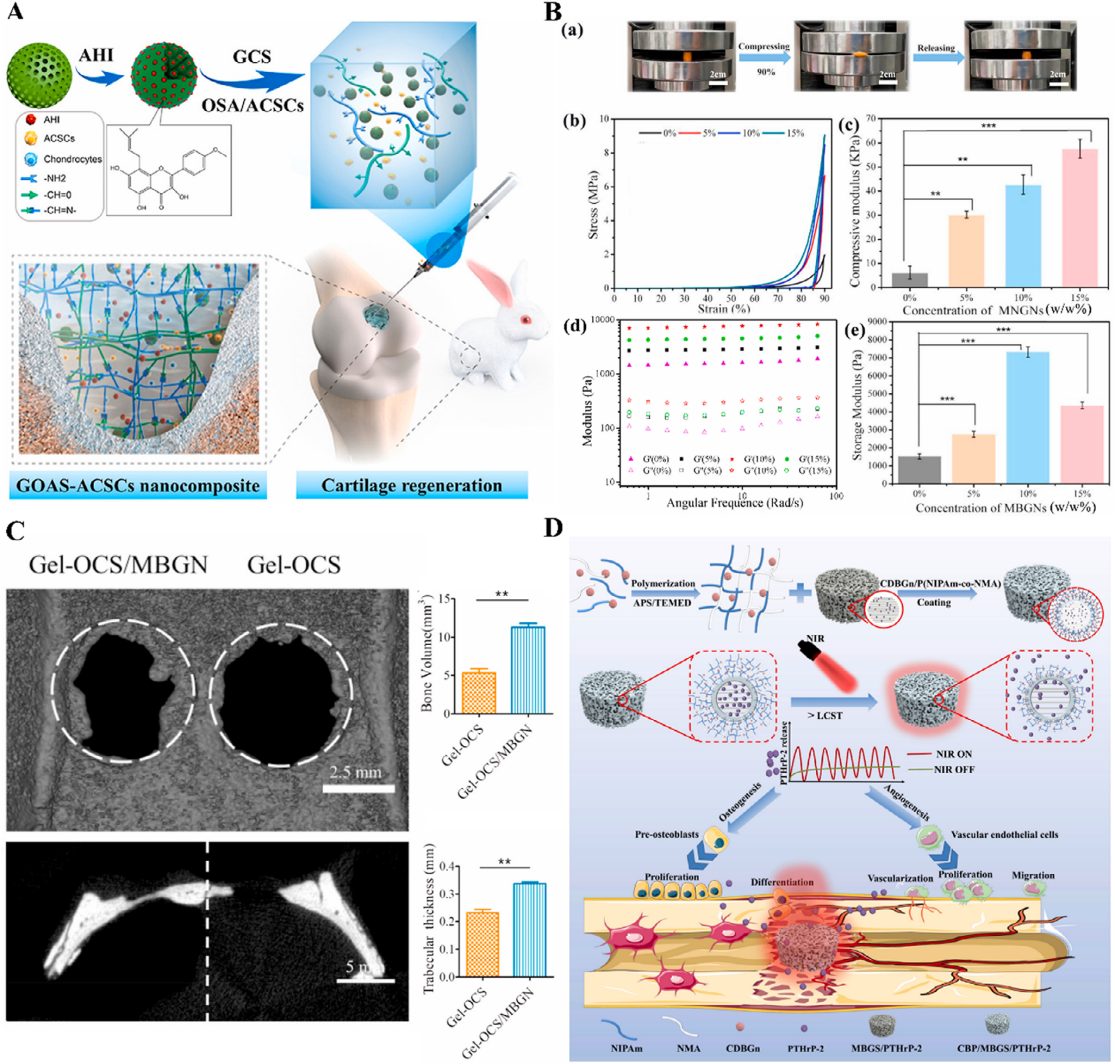
A) Illustration of the fabrication and cartilage regeneration application of injectable GOAS‐ACSCs engineered nanocomposites. Reproduced under the terms of the Creative Commons CC BY‐NC‐ND license.^[^ ^170^ ^]^ Copyright 2022, The Authors. B) Doping of MBGNs enhanced the mechanical properties of Gel‐OCS hydrogel. Reproduced under the terms of the Creative Commons CC BY‐NC‐ND license.^[^ ^171^ ^]^ Copyright 2020, The Authors. C) In vivo bone regeneration after 6 weeks of MBGNs‐doped Gel‐OCS hydrogel implantation. Reproduced under the terms of the Creative Commons CC BY‐NC‐ND license.^[^ ^171^ ^]^ Copyright 2020, The Authors. D) Schematic diagram of the synthesis and mechanism of CBP/MBGS/PTHrP‐2 bioscaffolds to regulate angiogenesis and osteogenesis to promote bone regeneration. Reproduced under the terms of the Creative Commons CC BY‐NC‐ND license.^[^ ^172^ ^]^ Copyright 2023, The Authors.

Hydrogels have been the focal material in tissue engineering in recent years. Their application in bone tissue engineering, combined with BMMs, fully exploits the complementary advantages, demonstrating multifaceted innovative breakthroughs. First, in the delivery of drugs and growth factors, the high specific surface area and ordered pore structure of mesoporous materials can efficiently load osteogenesis‐related factors (such as BMP‐2, VEGF) and antibiotics, while the 3D network structure of hydrogels achieves sustained controlled release through physical encapsulation. For example, Zhang et al. dispersed mesoporous silica loaded with Anhydroicaritin into chitosan hydrogel, which exhibited significant sustained and slow drug release for up to 10 weeks, with a cumulative release of 85.58 ± 2.37%, significantly promoting the chondrogenic differentiation of articular cartilage stem cells in cartilage defects (**Figure** **7**A).^[^ ^170^ ^]^ Second, the composite material can dramatically enhance mechanical compatibility. The Young's modulus of pure hydrogel is usually below 1 MPa, which is insufficient to meet the repair requirements of bone load‐bearing areas. However, incorporating 15 wt.% MBG nanoparticles into a hybrid gelatin/chondroitin sulfate hydrogel can increase the compressive strength to 9.05 MPa, approaching the mechanical range of cancellous bone (Figure 7B).^[^ ^171^ ^]^ Furthermore, the synergistic effect of the two can promote the reconstruction of the bone regeneration microenvironment. The MBG nanoparticles encapsulated by hydrogels can not only continuously release calcium, silicon, and phosphorus ions to promote osteogenesis, but also the nanoscale hydroxyapatite layer formed on their surface can achieve in situ integration with the host cancellous bone. Animal experiments showed that the composite material doubled the bone volume fraction in the rat cranial bone defect model at week 6 compared to pure hydrogel (Figure 7C).^[^ ^171^ ^]^ Intelligent responsive composite systems have attracted much attention in terms of functional expansion. For example, by modifying the surface of mesoporous materials with temperature‐sensitive polymers and combining them with photothermal‐responsive hydrogels, vascular formation‐osteogenesis sequential regulation can be achieved through NIR control. For instance, Liu et al. used a thermoresponsive hydrogel coating composed of N‐isopropylacrylamide (NIPAm) and N‐hydroxymethylacrylamide (NMA) to load PTH on MBG, and achieved precise control of the sustained and pulsatile release of PTHrP‐2 through external on/off NIR laser irradiation (Figure 7D).^[^ ^172^ ^]^ Benefiting from this dual‐mode release capability, the composite material produced satisfactory angiogenesis and bone repair effects both in vitro and in vivo.

**Figure 8:**
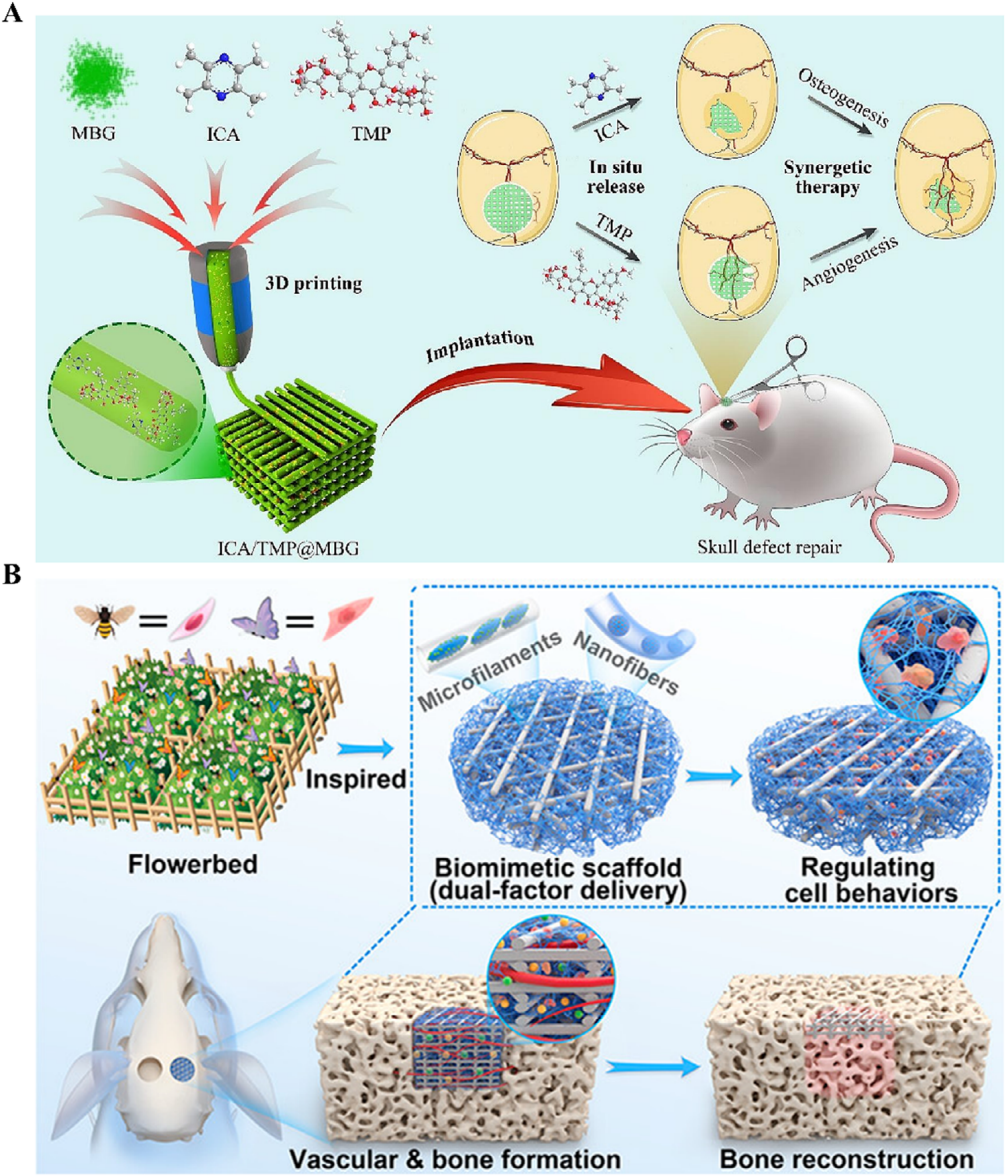
A) Schematic of 3D printed MBG scaffolds ICA/TMP@MBG for enhancing bone repair through synergistic angiogenesis and osteogenesis. Reproduced under the terms of the Creative Commons CC BY‐NC‐ND license.^[^ ^173^ ^]^ Copyright 2023, The Authors. B) Schematic of 3D printing combined with electrostatic spinning technology to fabricate DMSNs/SrHA@PGP scaffolds to deliver angiogenic drugs and osteogenic metal ions to enhance the repair of bone defects synergistically. Reproduced with permission.^[^ ^174^ ^]^ Copyright 2023, American Chemical Society.

3D printing technology has emerged as a revolutionary technique in bone tissue engineering in recent years. Bone regeneration scaffolds with complex microstructures can be fabricated by preparing BMMs into printing ink and utilizing extrusion printing technology. These 3D‐printed MBG scaffolds exhibit higher stability, enabling sustained and stable drug release. Wu et al. found that naringin or calcitonin gene‐related peptide (CGRP) co‐printed into MBG scaffolds showed stable, sustained release behavior for up to 21 days without initial burst release. In contrast, naringin and CGRP absorbed on the surface of MBG scaffolds were completely released within two days.^[^ ^175^ ^]^ In large segmental bone defects, meticulously designed 3D‐printed scaffolds can bridge tissue gaps and provide structural support to maintain physiological activities and cellular behaviors, such as nutrient transport, cell proliferation, differentiation, and maturation. Chen et al. integrated the osteoinductive icariin (ICA) and the angiogenic tetramethylpyrazine (TMP) into a 3D‐printed MBG scaffold to improve the treatment of large segmental bone defects (**Figure** **8**A).^[^ ^173^ ^]^ The composite scaffold gradually degrades to provide the necessary space for new bone tissue formation, and the in situ released ICA and TMP synergistically promote angiogenesis and osteogenesis. This composite scaffold exhibited better bone repair performance than scaffolds loaded with either ICA or TMP alone. Wa et al. also found that 3D‐printed MBG‐doped PCL scaffolds can participate in immune regulation and promote the osteogenic differentiation of BMSCs.^[^ ^176^ ^]^ Among them, PCL scaffolds with a fiber diameter of 500 µm, a pore size of 500 µm, and an MBG content of 10% have the best results. Moreover, combining 3D printing and electrospinning technologies enables the development of higher‐performance bone regeneration scaffolds based on BMMs. Zhou et al. combined these two technologies to develop a dual‐factor delivery scaffold with high mechanical strength, highly porous structure, and excellent angiogenic and osteogenic activity to treat large bone defects (Figure 8B).^[^ ^174^ ^]^ By assembling electrospun short nanofibers loaded with dimethyloxalylglycine (DMOG)‐loaded MSNs with a 3D‐printed strontium hydroxyapatite/poly(ε‐caprolactone) (SrHA@PCL) scaffold, a tunable porous structure can be easily achieved by varying the density of nanofibers, while the framework of SrHA@PCL ensures robust compressive strength. Due to the different degradation properties of electrospun nanofibers and 3D‐printed microwires, a sequential release behavior of DMOG and Sr ions was realized. In vitro and in vivo results indicated that the dual‐factor delivery scaffold possessed excellent biocompatibility and significantly promoted angiogenesis and osteogenesis by stimulating endothelial cells and osteoblasts.

Bone tissue engineering, a significant research direction in biomedical science, has achieved remarkable progress by integrating material science and biology. Key technologies such as biomimetic mineralization mechanisms, multifunctional scaffold design, vascularization strategies, and 3D printing technology have laid a solid foundation for bone regeneration engineering. Among them, the biomimetic mineralization mechanism, by simulating the natural bone mineralization process and utilizing the high specific surface area and tunable pore structure of mesoporous materials, has realized the oriented growth of hydroxyapatite, significantly enhancing the biocompatibility and mechanical properties of the materials. Multifunctional scaffold design, through the rational regulation of scaffold topology and functional integration, such as introducing drug molecules or bioactive factors into MBG scaffolds, has achieved on‐demand release and significantly improved bone regeneration outcomes. Vascularization strategies, leveraging the drug/ion loading capacity and controlled release characteristics of mesoporous materials, have accelerated vascularized bone regeneration by loading VEGF and its mimetic peptides. 3D printing technology, by preparing bioinks from biomaterials and using photopolymerization for printing, has enabled the fabrication of bone regeneration scaffolds with complex microstructures and stable drug release capabilities. Despite these advances, bone tissue engineering still faces numerous challenges, such as the long‐term biocompatibility of materials, precise control of in vivo degradation rates, and in‐depth elucidation of the mechanisms of multifactorial synergistic actions. Future research needs to optimize material design further and strengthen interdisciplinary cooperation to achieve more efficient and safer bone tissue regeneration strategies.

### 3.3 Bone Implant Coatings Against Infection

Biomaterial‐associated infection (BAI), an infection caused by microorganisms adhering to the implant surface, is a global health burden.^[^ ^177^ ^]^ It is commonly seen in orthopedic implants such as artificial joints, bone nails, and bone plates.^[^ ^178^, ^179^ ^]^ BAI can occur throughout the entire usage cycle of the implant. The main mechanisms of its occurrence include the adhesion and proliferation of bacteria on the implant surface and the formation of biofilms.^[^ ^180^ ^]^ Clinically, by altering the surface characteristics of bone implants, such as adding antibacterial coatings or designing antifouling surfaces, bacterial adhesion can be reduced or the formation of biofilms can be disrupted.^[^ ^181^ ^]^ As an ideal candidate material for the functional coating on the surface of bone implants, BMMs' hierarchical pore structure and programmable surface chemical properties provide innovative solutions to clinical problems associated with traditional metal implants, such as delayed osseointegration, bacterial biofilm formation, and long‐term foreign body reactions.^[^ ^182^ ^]^

**Figure 9:**
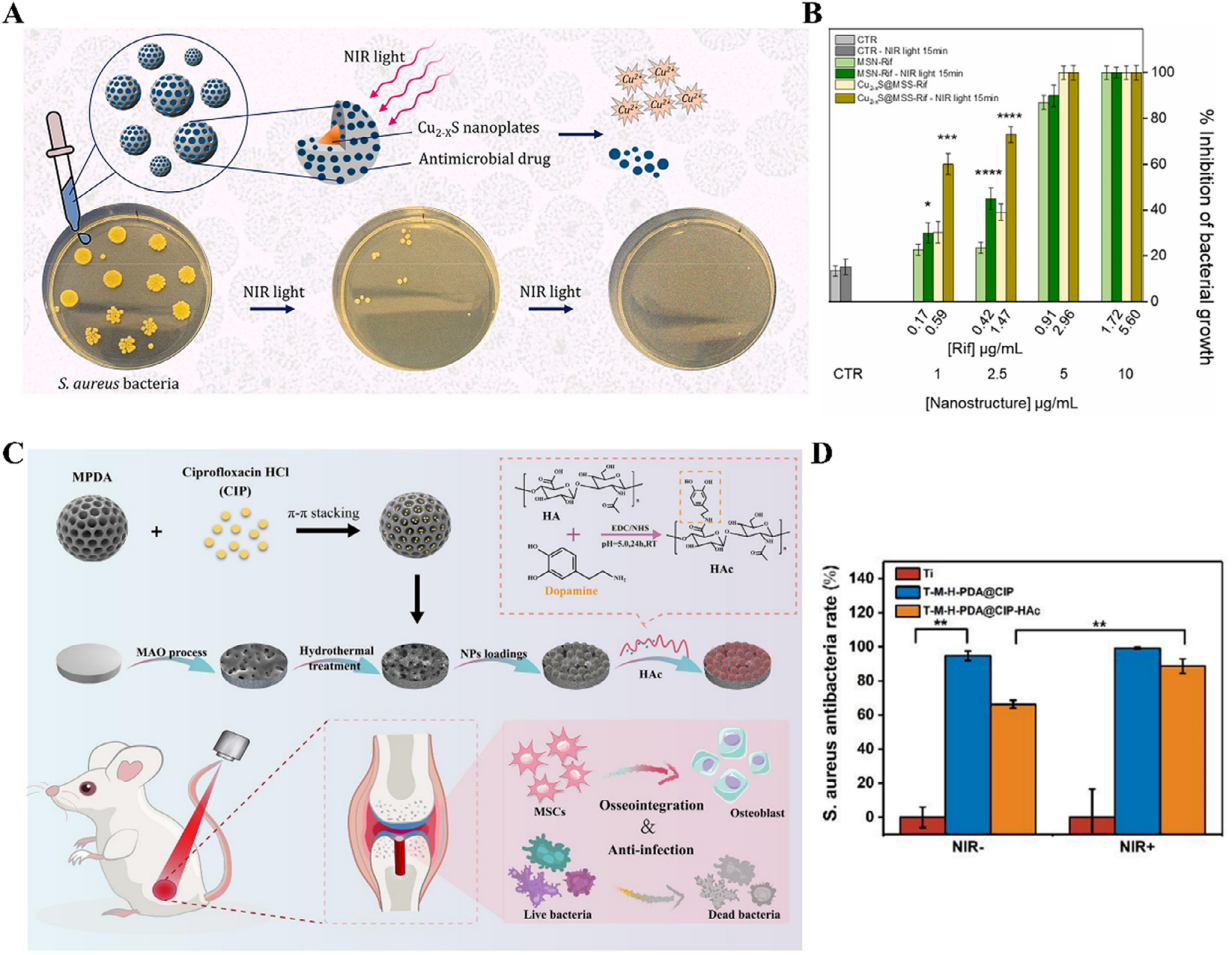
A) Schematic representation of Cu2‐xS@MSS loaded with antibiotic (levofloxacin or rifampicin) for local eradication of Gram‐positive S.aureus bacteria. Reproduced with permission.^[^ ^183^ ^]^ Copyright 2024, Elsevier. B) Evaluation of the antimicrobial effect of Cu2‐xS@MSS‐Rif. Reproduced with permission.^[^ ^183^ ^]^ Copyright 2024, Elsevier. C) Synergistic enhancement of titanium implants against bacterial infections based on photothermal and antibiotics. Reproduced with permission.^[^ ^184^ ^]^ Copyright 2023, Elsevier. D) Antimicrobial rate of Ti‐M‐H‐PDA@CIP‐HAc implants. Reproduced with permission.^[^ ^184^ ^]^ Copyright 2023, Elsevier.

The strategy of loading antimicrobial agents based on BMMs represents a classic approach for antibacterial coatings on bone implants. For instance, Tian et al. developed a gentamicin‐loaded mesoporous Fe_3_O_4_/carbonated hydroxyapatite coating‐modified Ti implant, demonstrating superior drug loading‐release properties to inhibit bacterial adhesion and prevent biofilm formation.^[^ ^185^ ^]^ Similarly, Nur Hidayatul et al. modified Ti implants with chlorhexidine‐loaded MBG, where the sustained release of antimicrobial agents effectively prevented Streptococcus mutans biofilm formation on the material surface.^[^ ^186^ ^]^ Unfortunately, direct antibiotic application on implants readily induces drug‐resistant bacterial strains that become increasingly difficult to eradicate. In pursuing novel antibacterial strategies, multi‐modal antimicrobial systems have emerged as promising solutions for treating such infections. Among these, the synergistic combination of photothermal antibacterial therapy and light‐responsive smart release of antimicrobial agents demonstrates optimal efficacy. For example, Federica et al. constructed a core@shell structure featuring Cu2‐xS triangular nanoplates as the core and mesoporous silica as the shell, further loaded with antimicrobial drugs. This design synergistically enhanced antibacterial effects by combining the inherent photothermal activity of Cu2‐xS nanoplates with pharmacological drug action (**Figure** **9**A).^[^ ^183^ ^]^ This combined system achieved ≈70% bacterial eradication when loaded with Rifampicin (Rif) and subjected to 15‐minute NIR irradiation at a concentration of 2.5 µgmL^−1^ (Figure 9B). To mitigate thermal damage to surrounding normal cells/tissues caused by high temperatures in photothermal therapy, Li et al. leveraged the synergy between mild photothermal effects and antibiotics under moderate‐temperature conditions with reduced drug dosage. They anchored ciprofloxacin‐loaded MPDA on titanium surfaces, coating them with sodium hyaluronate‐catechol (HAc) (Figure 9C).^[^ ^184^ ^]^ The hydrophilic HAc coating initially inhibited bacterial adhesion, while bacterial secretion of hyaluronidase triggered enzyme‐responsive degradation of HAc and antimicrobial release. NIR irradiation simultaneously activated the photothermal properties of MPDA nanoparticles and enhanced drug release. In vivo antibacterial experiments confirmed an 88.6% antibacterial rate for this functional coating (Figure 9D).

Doping metal ions with antibacterial properties is also an effective strategy for antibacterial coatings on the surface of BMMs bone implants. Commonly used metal ions include Cu, Ag, and Fe.^[^ ^187^ ^]^ For instance, Cui et al. utilized electrophoretic deposition technology to coat copper peptide GHK‐Cu (glycyl‐L‐histidyl‐L‐lysyl‐Cu^2^⁺)‐loaded MSNs onto titanium plates. The Cu^2^⁺ exhibited pH‐responsive release from the titanium surface, demonstrating excellent antibacterial effects by inhibiting bacterial growth or adhesion.^[^ ^188^ ^]^ Wang et al. reported that silver‐doped MBG‐coated titanium implant scaffolds displayed superior antibacterial properties.^[^ ^189^ ^]^ Antonio J. Salinas et al. reported that the synergistic antimicrobial effect of zinc ions in combination with curcumin enhances the bone regeneration properties of MBG.^[^ ^190^ ^]^ Although the individual or combined use of antibacterial agents or antibacterial metal ions can achieve remarkable antibacterial efficacy, their benefits during bone repair remain limited. Antibacterial‐osteogenic synergistic therapy has gradually emerged as a critical research direction for bone implant coatings, aiming to develop novel strategies that simultaneously enable efficient antibacterial action and promote bone tissue repair and regeneration.^[^ ^191^ ^]^ Among these advancements, key technologies such as metal ion synergy and photothermal antibacterial multifunctional systems have laid a solid foundation for antibacterial‐osteogenic synergistic therapy.

**Figure 10:**
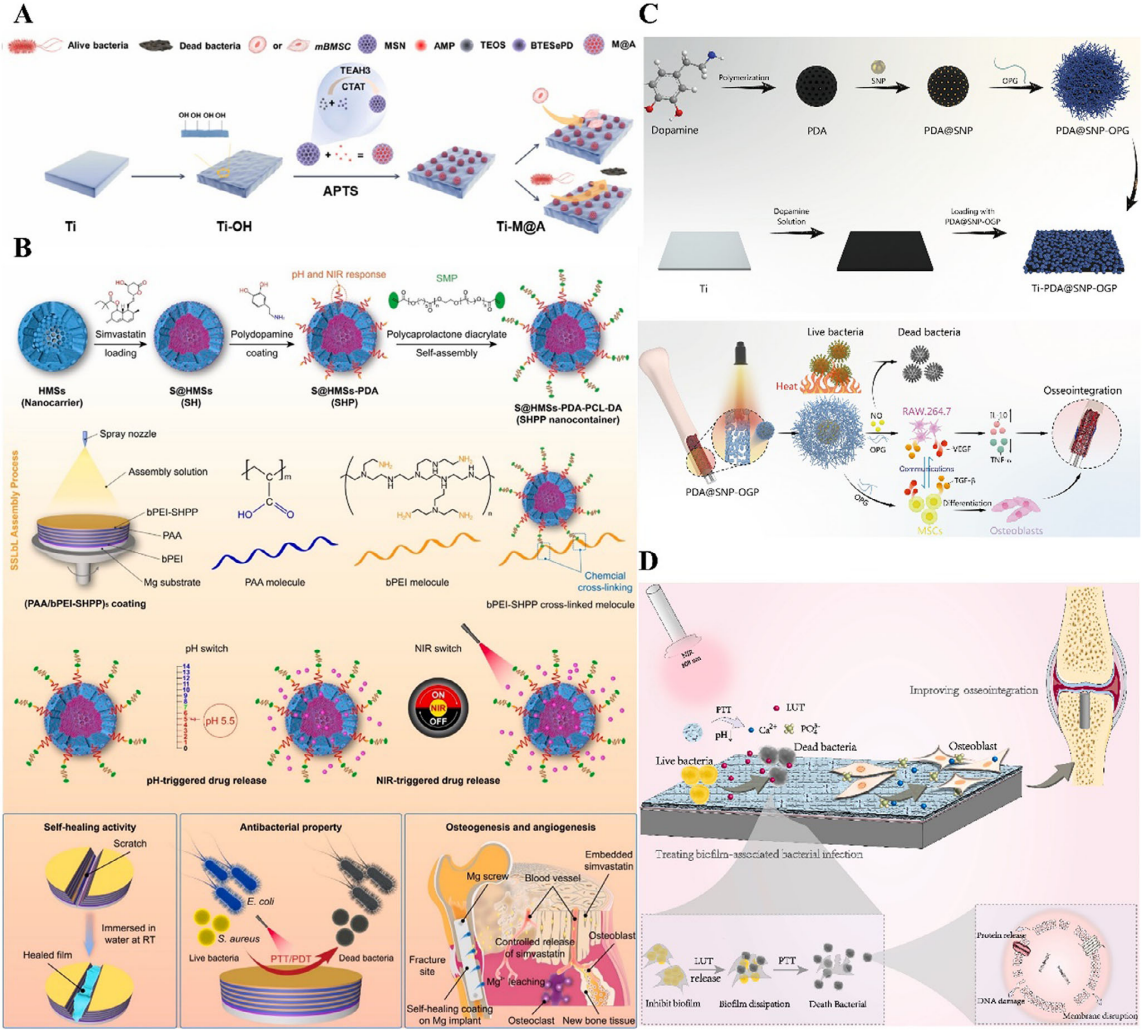
A) Antimicrobial peptide diselenide‐bridged MSNs functionalized titanium implants enhance inflammation and modulation of osseointegration. Reproduced with permission.^[^ ^32^ ^]^ Copyright 2024, Elsevier. B) pH/NIR‐responsive and self‐repairing coating of modified magnesium alloys endows them with bactericidal, osteogenic, and angiogenic properties. Reproduced with permission.^[^ ^194^ ^]^ Copyright 2023, Elsevier. C) Eliminating methicillin‐resistant Staphylococcus aureus biofilm from titanium implants by photothermally triggering nitric oxide and immunotherapy to enhance osseointegration. Reproduced under the terms of the Creative Commons CC BY license.^[^ ^195^ ^]^ Copyright 2023, The Authors. D) Schematic illustration of combining photothermal therapy and population‐sensing inhibition strategies to improve osseointegration and treat biofilm‐associated bacterial infections on Ti‐based implants. Reproduced under the terms of the Creative Commons CC BY‐NC‐ND license.^[^ ^196^ ^]^ Copyright 2022, The Authors.

The synergistic effect of metal ions serves as one of the core strategies in antibacterial‐osteogenic dual‐functional therapy for bone implant coatings. Silver ions (Ag⁺) are widely used in medical materials due to their potent antibacterial properties, while strontium ions (Sr^2^⁺) and copper ions (Cu^2^⁺) have garnered significant attention for their ability to promote vascularized bone regeneration. In recent years, researchers have focused on developing material systems that harness the synergistic effects of these two types of metal ions. Among them, BMMs have emerged as ideal carriers due to their excellent physicochemical properties. For instance, Guo et al. demonstrated that a polyelectrolyte multilayer coating containing silver/strontium‐doped MBG on 316L stainless steel enables sustained release of both ions, achieving efficient antibacterial effects while promoting bone tissue repair and regeneration.^[^ ^192^ ^]^ Jiang et al. also reported that a coating incorporating silver/copper co‐doped MSNs and a chitosan adhesive exhibits robust antibacterial and pro‐angiogenic capabilities.^[^ ^193^ ^]^ Furthermore, the synergy between osteogenesis‐promoting metal ions and antibacterial agents plays a pivotal role in dual‐functional antibacterial‐osteogenic therapy. A notable example is the work by Dong et al., who developed a functionalized titanium implant (Ti‐M@A) by immobilizing diselenide‐bridged MSNs loaded with the antimicrobial peptide HHC36. This system achieved sustained release of the peptide for over 30 days, demonstrating >95.71% antibacterial efficacy against four clinical pathogens (Staphylococcus aureus, Escherichia coli, Pseudomonas aeruginosa, and methicillin‐resistant Staphylococcus aureus [MRSA]). Concurrently, selenium (Se) in the MSNs significantly enhanced osteogenic differentiation of BMSCs (**Figure** **10**A).^[^ ^32^ ^]^ Such multi‐level regulatory strategies provide novel insights for developing high‐performance dual‐functional materials with combined antibacterial and osteogenic properties.

The multifunctional photothermal antibacterial system is another important research direction in the dual‐effect treatment of antibacterial and osteogenic properties for bone implant coatings. After absorbing light of a specific wavelength (such as near‐infrared), photothermal materials can efficiently convert light energy into heat energy, raising the local temperature. This process destroys the cell structure of bacteria, leading to their death. By introducing osteogenic drugs into the photothermal material system, intelligent drug release triggered by photothermal means can be achieved, maintaining a relatively high drug concentration locally. This further enhances the antibacterial effect and promotes bone tissue regeneration. For example, Zhao et al. constructed a self‐healing polymer coating on a magnesium alloy containing mesoporous silica nanospheres with photothermal antibacterial properties and loaded with simvastatin (Figure 10B).^[^ ^194^ ^]^ Here, the drug‐loaded mesoporous silica nanospheres were modified with double layers of polydopamine and polycaprolactone diacrylate, endowing them with the ability of pH‐ and NIR‐responsive drug release. Experimental results showed that the coating had good photothermal antibacterial properties under NIR irradiation. The release of simvastatin in a pH‐ and NIR‐responsive manner demonstrated enhanced angiogenesis and osteogenic effects. In addition, combining photothermal antibacterial therapy with gas antibacterial therapy can achieve better antibacterial and osteogenic effects. For instance, Yu et al. coated a Ti implant with mesoporous polydopamine nanoparticles and loaded it with a nitric oxide (NO) release donor, sodium nitroprusside (SNP), and an osteogenic growth peptide (OGP). They achieved the controlled release of NO and OGP through NIR laser‐stimulated photothermal conversion of PDA (Figure 10C).^[^ ^195^ ^]^ The combination of gas therapy and photothermal therapy significantly improved the antibacterial effect of the bone implant, eradicating the methicillin‐resistant Staphylococcus aureus (MRSA) biofilm. Combined with the osteogenic growth peptide (OGP), bone bonding is significantly enhanced. To solve the problem that the high temperature of photothermal therapy may damage the surrounding normal cells/tissues, Hu et al. proposed surface modification of a titanium substrate by combining photothermal therapy and the quorum‐sensing inhibition strategy. This approach improved bone bonding and treatment of biofilm‐related bacterial infections under low‐temperature conditions (Figure 10D).^[^ ^196^ ^]^ Calcium phosphate (CaP) was wrapped with MPDA nanoparticles loaded with luteolin (LUT) and then used to modify the Ti implant further. As a quorum‐sensing inhibitor, luteolin (LUT) can be triggered to release in the acidic environment of bacterial biofilm infection, effectively inhibiting/disrupting the bacterial biofilm. Under NIR irradiation, hyperthermia induced by the photothermal conversion effect of MPDA destroyed the integrity of the bacterial membrane, synergistically causing protein leakage and a decrease in ATP levels. By combining photothermal therapy and the quorum‐sensing inhibition strategy, the surface‐functionalized titanium substrate achieved an antibacterial rate of over 95.59% against Staphylococcus aureus and a clearance rate of up to 90.3% for the formed biofilm, thus realizing high‐efficiency treatment of bacterial biofilm infections at low temperatures. Moreover, the release of Ca^2^⁺ and PO_4_^3−^ accelerated cell growth and bone tissue healing. These multifunctional integrated design concepts provide a new solution for developing high‐performance photothermal antibacterial and osteogenic materials.

Due to their high recurrence rate and drug resistance risk, BAI has become a core challenge in the clinical application of orthopedic implants. Traditional strategies, centered on antibacterial coatings, inhibit biofilm formation by loading antibiotics (such as gentamicin and chlorhexidine) or metal ions (Cu^2^⁺, Ag⁺) into BMMs. However, their single antimicrobial mechanism is prone to inducing drug resistance and neglects the dynamic needs of bone repair. To address these issues, researchers have turned to multimodal synergistic strategies that integrate photothermal effects, metal ion synergies, and intelligent responsive release systems to balance antimicrobial and osteogenic functions. Photothermal antimicrobial systems (such as Cu_2‐x_S core‐shell structures and polydopamine nanoparticles) can efficiently eliminate biofilms by triggering local temperature increases and controlled drug release through near‐infrared stimulation. However, the issue of thermal damage remains controversial. Subsequently, a mild solution combining low‐temperature photothermal effects with antibiotics has been optimized. Through dual‐functional design, metal ion synergy strategies (such as Ag/Sr and Cu/Se co‐doping) achieve synergistic antibacterial and pro‐angiogenic/osteogenic effects. However, the ion release kinetics and long‐term biocompatibility still require systematic evaluation. Moreover, the innovative combination of gas therapy (NO) and quorum sensing inhibition (luteolin) provides a new direction for low‐temperature, efficient antibiofilm activity and promoting bone integration. However, the clinical translation of this approach needs to overcome the controllability issues of complex DDS. Current research has broken through the limitations of single functionality but primarily relies on in vitro or small animal models. It lacks precise responsiveness to the heterogeneity of the infection microenvironment (such as pH and enzyme dynamics), and the temporal matching mechanism of antimicrobial‐osteogenic efficacy remains unclear. In the future, there is a need for a deep integration of materials engineering, microbiology, and regenerative medicine to develop novel intelligent coatings that dynamically adapt to the implantation cycle, to break through the bottleneck of drug resistance, and achieve precise synergy between infection control and functional reconstruction.

### 3.4 Bone Disease Theranostics

Theranostics (integrated diagnosis and treatment) has emerged as a critical research direction in the biomedical field in recent years. It aims to develop novel strategies for precise diagnosis and efficient therapy by integrating materials science and medical technologies.^[^ ^197^, ^198^, ^199^ ^]^ Key advancements in multimodal imaging and visualized therapy have laid a robust foundation for theranostics in bone‐related diseases.

**Figure 11:**
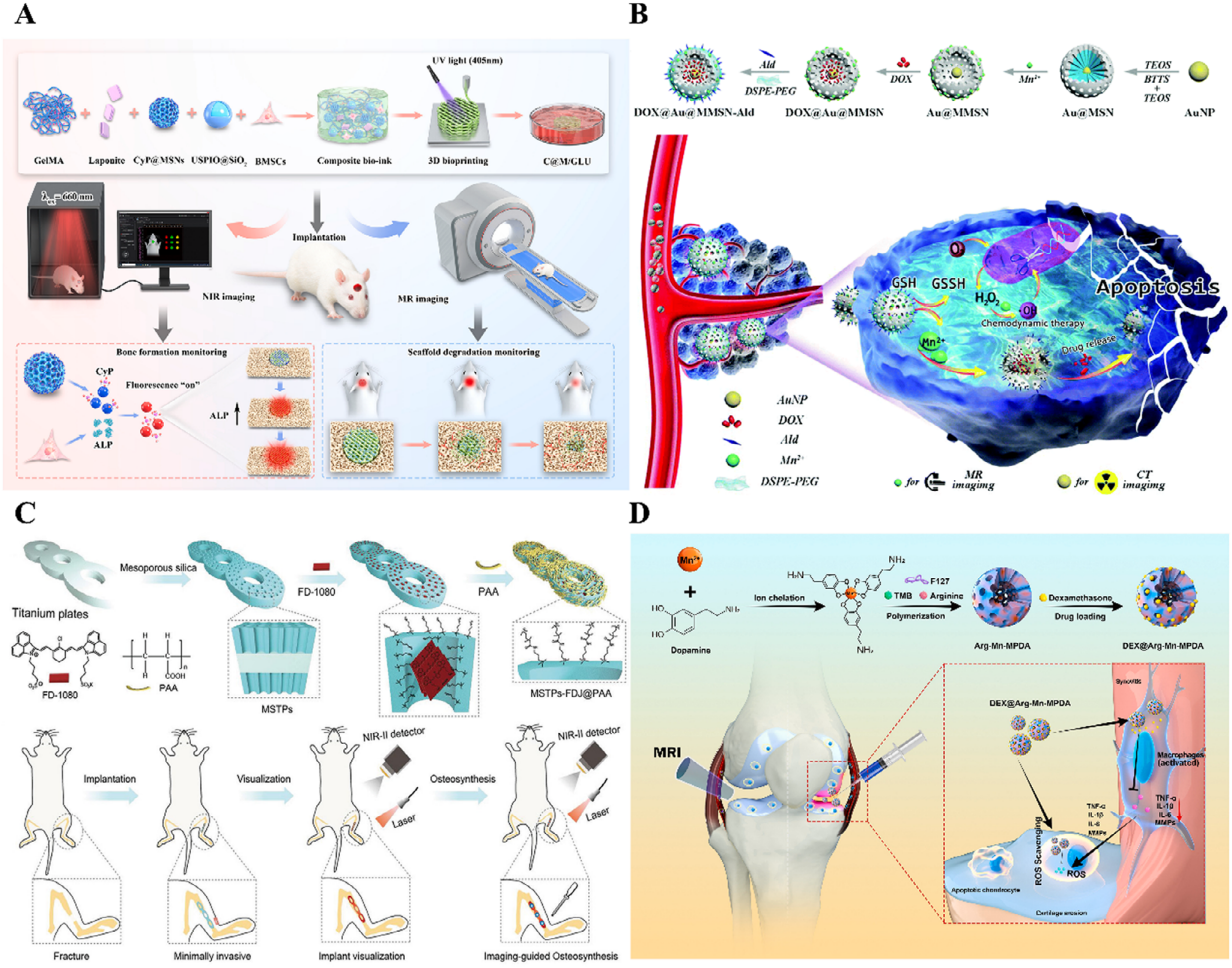
A) Schematic of NIR‐FL/MR dual‐modality imaging bioscaffold design and its use for noninvasive in situ monitoring of bone repair. Reproduced under the terms of the Creative Commons CC BY‐NC‐ND license.^[^ ^33^ ^]^ Copyright 2024, The Authors. B) Schematic of preparing manganese‐doped gold‐core mesoporous silica particles and their use as a combined dual‐modality imaging and chemo‐chemical kinetics treatment for osteosarcoma. Reproduced with permission.^[^ ^203^ ^]^ Copyright 2020, Royal Society of Chemistry. C) Schematic of preparing a mesoporous silica‐coated titanium plate labeled by an NIR‐II J aggregation and its use for MSTP‐based NIR‐II imaging‐guided osteosynthesis. Reproduced with permission.^[^ ^204^ ^]^ Copyright 2021, John Wiley and Sons. D) Schematic diagram of mesoporous polydopamine nanoprobes that can be used for MRI visualization of osteoarthritis diagnosis and treatment. Reproduced with permission.^[^ ^35^ ^]^ Copyright 2023, Elsevier.

Multimodal imaging is one of the core concepts in theranostics. As two crucial biomedical imaging techniques, magnetic resonance imaging (MRI) and fluorescence (FL) imaging have attracted extensive attention due to their complementary advantages. Researchers have been dedicated to developing material systems capable of achieving MRI and FL dual‐modal imaging in recent years. Using dual‐modal imaging technology to track the degradation of bone tissue engineering scaffolds in real‐time and accurately will enhance the refinement of scaffold materials and designs, which is beneficial for achieving optimal bone regeneration.^[^ ^200^, ^201^, ^202^ ^]^ Based on this, He et al. designed a multimodal NIR‐FL/MR imaging biologic scaffold for non‐invasive in‐situ monitoring of bone repair (**Figure** **11**A).^[^ ^33^ ^]^ This scaffold was fabricated by 3D bioprinting a composite bioink composed of MSNs loaded with CyP (a hemicyanine NIR‐FL probe that interacts with alkaline phosphatase (ALP) to emit fluorescence), silica‐coated USPIO (an MR contrast agent), gelatin methacrylate (GelMA), and lapis lazuli. With the secretion of ALP during early osteogenesis, the CyP released from the integrated scaffold interacts with ALP, enhancing fluorescence intensity. Subsequently, the early osteogenic activity can be predicted through fluorescence image acquisition. Meanwhile, the integration of USPIO into the scaffold endows it with MR imaging capabilities, which is expected to enable the observation of scaffold degradation at different stages. In addition, the research on multimodal imaging also involves the integration of multiple functions. For example, by introducing the function of sustained drug release into mesoporous materials, local treatment can be achieved simultaneously with accurate diagnosis. Zhou et al. constructed a dual‐modal imaging and chemo‐chemodynamic combined therapy nanoplatform for osteosarcoma, Au@MMSN‐Ald, by in situ doping metal manganese into gold‐core MSNs and modifying them with alendronate (Figure 11B).^[^ ^203^ ^]^ The Au nanoparticles in the core and the manganese ions released from the shell enable CT and MR dual‐modal imaging to verify the effective accumulation of Au@MMSN‐Ald at the tumor site. The DOX‐loaded Au@MMSN‐Ald exhibits excellent tumor microenvironment‐responsive drug release efficiency. This multifunctional integrated design concept provides a brand‐new solution for theranostics.

Visualized therapy is another important research direction in integrating diagnosis and treatment. The mesoporous material coating endows bone implants with an excellent pore‐channel structure, which can load specific markers for the precise navigation of implant surgery, effectively reducing surgical wounds and shortening the operation time. For example, Sun et al. reported a novel biocompatible NIR‐II J‐polymer‐labeled mesoporous silica‐coated titanium plate for imaging‐guided bone synthesis with minimal invasion (Figure 11C).^[^ ^204^ ^]^ The NIR‐II cyanine dye FD‐1080 is loaded into the mesoporous channels. By adjusting the polarity of the solvent, the FD‐1080 dye can form uniform J‐aggregates with a maximum absorption and emission wavelength exceeding 1300 nm. Meanwhile, the mesoporous implant labeled with biocompatible NIR‐II J‐aggregates has a relatively deep tissue penetration depth and high biological imaging resolution. More importantly, the NIR‐II fluorescence of the implantable steel plate can guide osteogenesis with minimal surgical trauma and operation time. In addition, mesoporous polydopamine nanoprobes can be used for MRI‐visualized diagnosis and treatment of osteoarthritis. Liu et al. doped arginine (Arg) and manganese ions (Mn^2+^) into mesoporous polydopamine, endowing it with excellent anti‐ROS and MRI‐visualized probe functions. They further loaded DEX to construct the DEX@Arg‐Mn‐MPDA (DAMM) nanodiagnostic and therapeutic probe, which can simultaneously inhibit the inflammatory response of synovial macrophage polarization and the apoptosis of articular chondrocytes, thereby delaying the development of OA (Figure 11D).^[^ ^35^ ^]^ At the same time, this diagnostic and therapeutic probe has T1‐T2 dual‐mode MRI contrast ability, which is expected to realize MRI‐visualized intra‐articular drug delivery, effectively monitor the drug concentration in the pathological microenvironment, and accurately diagnose the development process of OA.

**Figure 12:**
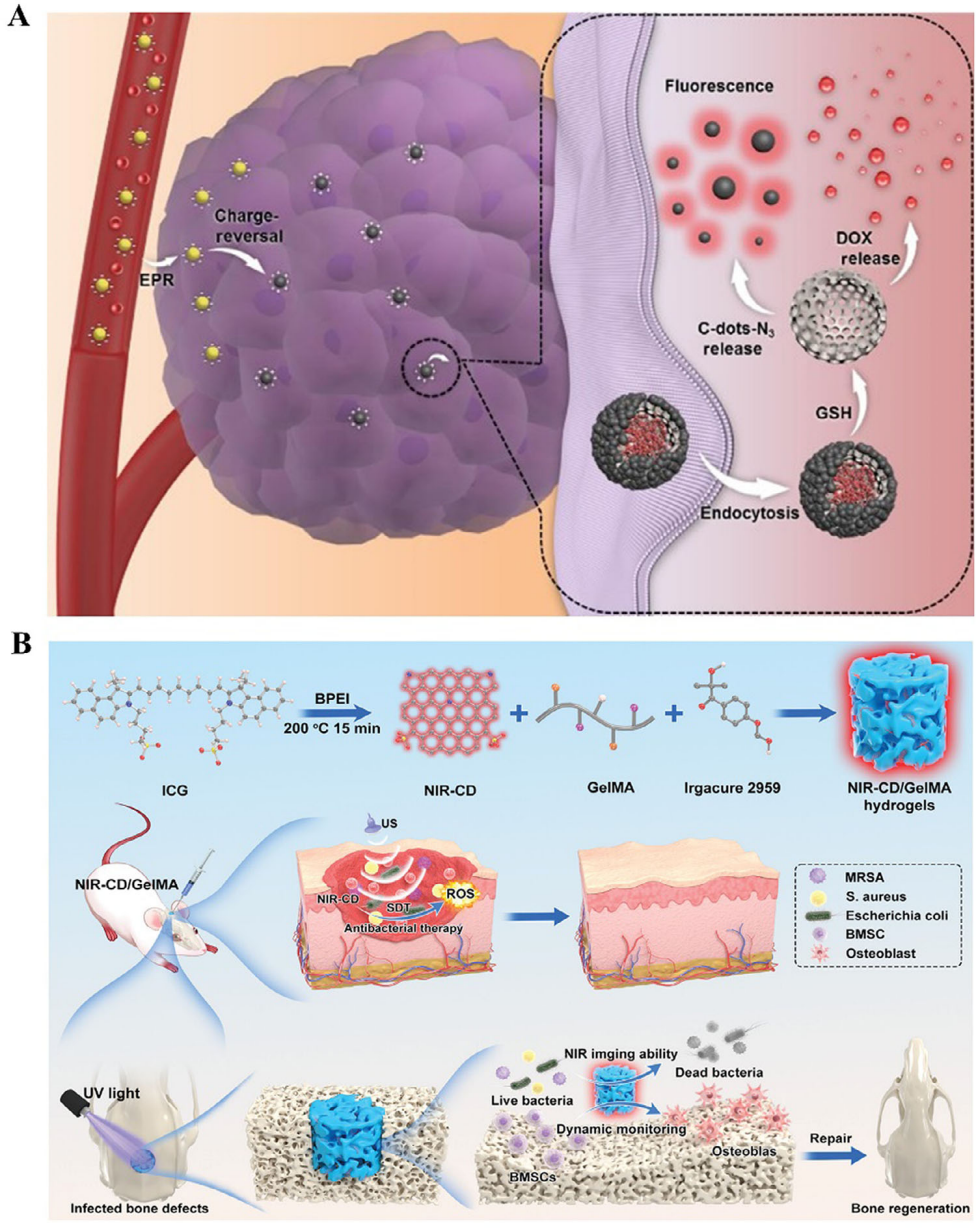
A) Schematic of a dual stimulus‐responsive NIR‐emitting carbon dots/hollow mesoporous silica‐based integrated therapeutic diagnostics platform for real‐time visualization of drug delivery. Reproduced with permission.^[^ ^205^ ^]^ Copyright 2021, Tsinghua University Press and Springer‐Verlag GmbH Germany, part of Springer Nature. B) Schematic of NIR carbon dots with antimicrobial and osteogenic activity for acoustic kinetic treatment of infected bone defects. Reproduced with permission.^[^ ^206^ ^]^ Copyright 2024, John Wiley and Sons.

As an ideal fluorescent nanomaterial, carbon dots (CDs) have great application potential in the integrated diagnosis and treatment of bone‐related diseases due to their adjustable fluorescence properties, good biocompatibility, and simple surface functionalization. They can serve as fluorescent probes to visualize drug release and monitor various metabolic processes in real‐time. For example, Chen et al. modified CDs onto the surface of mesoporous silica loaded with DOX via disulfide bonds. This modification can prevent DOX leakage and serve as a fluorescent imaging agent to visualize drug release (**Figure** **12**A).^[^ ^205^ ^]^ When the carrier enters tumor cells, the disulfide bonds are reduced by intracellular glutathione (GSH), leading to the release of DOX into the cells and the release of carbon dots, which then produce fluorescence. The results of in vitro and in vivo experiments show that the nanocarrier has a good therapeutic effect, no apparent side effects, real‐time fluorescent imaging ability of drug metabolism, and can provide timely feedback on the treatment effect. In addition, CDs also show significant effects in sonodynamic therapy due to their excellent photosensitive properties. For example, Tang et al. synthesized NIR fluorescent CDs (NIR‐CD) with osteogenic differentiation activity and sonodynamic antibacterial properties to visually repair infected bone defects (Figure 12B).^[^ ^206^ ^]^ Due to sulfonic acid acceptors and pyridine nitrogen donors, NIR‐CD exhibits enhanced sonodynamic activity of the p‐n junction and NIR imaging ability promoted by a narrow bandgap. It can act as an NIR sonosensitizer to generate ROS under ultrasound stimulation to eradicate methicillin‐resistant Staphylococcus aureus (MRSA). Because of the abundant sulfonic acid groups, NIR‐CD shows strong negative charge characteristics and excellent osteogenic activity. This integrated diagnosis‐treatment model enables the efficient killing of pathogens and promotes the repair and regeneration of bone tissue.

While these integrated theranostic platforms demonstrate remarkable innovation in bone disease management, their clinical translation faces a fundamental constraint: the inherent limitations of photothermal penetration depth. Current NIR‐based systems achieve tissue penetration of 3–8 mm—sufficient for superficial lesions but critically inadequate for targeting deep‐seated pathological bone structures such as vertebral bodies or femoral heads. This depth barrier introduces significant therapeutic uncertainty, as heterogeneous bone mineral density (30–90% variance in osteoporosis) creates unpredictable photon scattering and energy attenuation. Consequently, localized hyperthermia may fail to reach therapeutic thresholds (>42 °C) at clinically relevant depths, risking sublethal tumor cell survival in osteosarcoma or insufficient anti‐inflammatory effects in OA. Future designs must prioritize wavelength optimization (e.g., shifting to 1500–1700 nm NIR‐IIb window), incorporate real‐time thermal feedback systems, and develop combinatorial approaches with ultrasound or radiofrequency augmentation to overcome this biophysical frontier.

## 4 Conclusion and Future Perspectives

With the intensification of global population aging, bone‐related diseases have emerged as one of the most severe public health challenges in the 21st century. Issues such as osteoporosis, bone defects, and bone infections not only compromise patients' quality of life but also impose substantial economic burdens on healthcare systems. In this context, BMMs have become a research hotspot in bone repair due to their unique physicochemical characteristics, including high specific surface area, tunable pore size, excellent biocompatibility, and multifunctional integration capabilities. In bone‐targeted delivery systems, surface functionalization modifications have enabled efficient drug accumulation and controlled release at lesion sites; the mesoporous structural design of tissue engineering scaffolds simultaneously satisfies mechanical support and cellular regulation requirements; optimized implant coatings significantly enhance antibacterial performance and osseointegration efficiency; while integrated diagnostic‐therapeutic systems break the temporal‐spatial barriers between diagnosis and treatment of bone‐related diseases. These advancements collectively drive a paradigm shift in bone disease management strategies from macro‐intervention to micro‐precision regulation.

Despite the promising preclinical advancements in BMMs for bone repair, significant translational challenges must be addressed before clinical adoption. First, scalable manufacturing remains a critical bottleneck. Reproducing complex mesoporous architectures (e.g., ordered pore symmetry, hierarchical structures) across industrial batches requires stringent control of template‐directed synthesis parameters. Variations in precursor ratios, pH, or thermal conditions during evaporation‐induced self‐assembly (EISA) can compromise material consistency, a key regulatory requirement for therapeutic nanomaterials. Second, long‐term biosafety profiles necessitate a comprehensive evaluation. While BMMs exhibit excellent biocompatibility in short‐term studies, potential accumulation of degradation byproducts (e.g., silicic acid from mesoporous silica, metal ions from MOFs/bioglasses) in bone marrow microenvironments raises concerns about chronic toxicity and immunogenicity. Finally, evolving regulatory frameworks (FDA Nanotechnology Guidance, NMPA Technical Guidelines for Nanomedicine) demand rigorous characterization of nanoparticle sterility, endotoxin levels, batch‐to‐batch variability, and biodistribution kinetics – hurdles compounded by the structural complexity of engineered BMMs. Addressing these challenges requires interdisciplinary collaboration between materials scientists, toxicologists, and regulatory specialists to establish standardized production protocols and safety assessment methodologies.

Shortly, the field urgently requires building stronger bridges between fundamental material research and clinical translation. More research on mesoporous materials will focus on bone matrix component‐mimicking materials such as mesoporous hydroxyapatite, calcium phosphate, and bioactive glass. Mesoporous materials' synthesis and functional design need to transition from traditional empirical approaches to data‐ and intelligence‐driven innovation. By constructing material genome databases and machine learning models, artificial intelligence could enable high‐throughput virtual screening of template‐precursor combinations, accurately predict mesophase formation trends and pore topology evolution patterns, significantly shortening new material development cycles. Reinforcement learning‐based inverse design systems can decode nonlinear mapping relationships between synthesis parameters (e.g., pH value, ionic strength) and target properties (pore size distribution, channel structure), guiding efficient preparation of customized mesoporous carriers.

Future research on DDS will emphasize intelligent and synergistic therapeutic capabilities. On one hand, incorporating multimodal stimulus‐responsive mechanisms (e.g., light, ultrasound, magnetic fields) could achieve precise spatiotemporal control of drug release to enhance therapeutic efficacy. Meanwhile, developing multifunctional delivery systems co‐loaded with anti‐resorptive and pro‐osteogenic drugs may realize synergistic therapeutic effects, substantially improving bone repair efficiency. Additionally, enhanced targeting specificity and bioavailability will be crucial. Surface modification with specific targeting molecules (peptides, antibodies, glycans) enables precise bone tissue recognition and targeted delivery, increasing drug concentration in target tissues while reducing off‐target distribution. Standardized biomanufacturing processes must be established for clinical translation, particularly addressing long‐term metabolic tracking challenges through technologies like isotope labeling for dynamic nanoparticle tracing in bone marrow cavities.

Simultaneously, mesoporous materials' hierarchical structural editability and biological function programmability position them as core enabling tools to overcome bone organoid technology bottlenecks. Macro‐mesoporous dual‐network scaffolds constructed via 3D printing combined with in situ mineralization strategies not only mimic natural bone matrix's mechanical transduction properties but also achieve spatiotemporal‐controlled growth factor release through mesoporous confinement effects, providing dynamic biochemical microenvironments for vascularized bone organoid self‐organization. Smart hydrogel composites with tunable elastic modulus leverage external field‐responsive characteristics (e.g., piezoelectric effects) to dynamically regulate matrix stiffness, precisely replicating stage‐specific mechanical microenvironment evolution during bone repair to direct stem cell fate determination. For organoid functional upgrading, mesoporous carrier‐loaded neurovascular co‐regulatory factors enable synchronous vascular network formation and nerve terminal infiltration through sequential release patterns, reconstructing multicellular communication networks in bone tissue.

Future integrated diagnostic‐therapeutic systems will emphasize multifunctional integration and clinical applicability. Combining multimodal imaging technologies (MRI, fluorescence imaging) could enable real‐time monitoring of bone scaffold degradation and repair processes to optimize material design and treatment protocols. Concurrently, implant surface modification with specific markers may achieve precise surgical navigation, reducing operative trauma while enhancing therapeutic outcomes.

## Conflict of Interest

The authors declare no conflict of interest.
